# Hemispheric asymmetries in resting‐state EEG and fMRI are related to approach and avoidance behaviour, but not to eating behaviour or BMI

**DOI:** 10.1002/hbm.24864

**Published:** 2019-11-21

**Authors:** Filip Morys, Lieneke K. Janssen, Elena Cesnaite, Frauke Beyer, Isabel Garcia‐Garcia, Jana Kube, Deniz Kumral, Franziskus Liem, Nora Mehl, Keyvan Mahjoory, Anne Schrimpf, Michael Gaebler, Daniel Margulies, Arno Villringer, Jane Neumann, Vadim V. Nikulin, Annette Horstmann

**Affiliations:** ^1^ Leipzig University Medical Centre IFB Adiposity Diseases Leipzig Germany; ^2^ Department of Neurology Max Planck Institute for Human Cognitive and Brain Sciences Leipzig Germany; ^3^ Montreal Neurological Institute McGill University Montreal Quebec Canada; ^4^ Subproject A1/A5, CRC1052 “Obesity Mechanisms” University of Leipzig Leipzig Germany; ^5^ Brandenburg University of Technology Cottbus‐Senftenberg Cottbus Germany; ^6^ MindBrainBody Institute at the Berlin School of Mind and Brain Humboldt‐Universitaet zu Berlin Berlin Germany; ^7^ University Research Priority Program “Dynamics of Healthy Aging” University of Zurich Zurich Switzerland; ^8^ Max Planck Research Group for Neuroanatomy & Connectivity Max Planck Institute for Human Cognitive and Brain Sciences Leipzig Germany; ^9^ Faculty of Psychology Technical University Dresden Dresden Germany; ^10^ University of Muenster, Institute for Biomagnetism and Biosignal Analysis Muenster Germany; ^11^ Brain and Spine Institute Paris France; ^12^ Ernst‐Abbe‐Hochschule – University of Applied Sciences Jena Germany; ^13^ Centre for Cognition and Decision Making Institute for Cognitive Neuroscience, National Research University Higher School of Economics Moscow Russia; ^14^ Department of Neurology Charité – Medical University Berlin Berlin Germany; ^15^ Department of Psychology and Logopedics, Faculty of Medicine University of Helsinki Helsinki Finland

**Keywords:** approach/avoidance behaviour, BMI, EEG, fMRI, hemispheric asymmetries, obesity, resting‐state

## Abstract

Much of our behaviour is driven by two motivational dimensions—approach and avoidance. These have been related to frontal hemispheric asymmetries in clinical and resting‐state EEG studies: Approach was linked to higher activity of the left relative to the right hemisphere, while avoidance was related to the opposite pattern. Increased approach behaviour, specifically towards unhealthy foods, is also observed in obesity and has been linked to asymmetry in the framework of the right‐brain hypothesis of obesity. Here, we aimed to replicate previous EEG findings of hemispheric asymmetries for self‐reported approach/avoidance behaviour and to relate them to eating behaviour. Further, we assessed whether resting fMRI hemispheric asymmetries can be detected and whether they are related to approach/avoidance, eating behaviour and BMI. We analysed three samples: Sample 1 (*n* = 117) containing EEG and fMRI data from lean participants, and Samples 2 (*n* = 89) and 3 (*n* = 152) containing fMRI data from lean, overweight and obese participants. In Sample 1, approach behaviour in women was related to EEG, but not to fMRI hemispheric asymmetries. In Sample 2, approach/avoidance behaviours were related to fMRI hemispheric asymmetries. Finally, hemispheric asymmetries were not related to either BMI or eating behaviour in any of the samples. Our study partly replicates previous EEG findings regarding hemispheric asymmetries and indicates that this relationship could also be captured using fMRI. Our findings suggest that eating behaviour and obesity are likely to be mediated by mechanisms not directly relating to frontal asymmetries in neuronal activation quantified with EEG and fMRI.

## 1 INTRODUCTION

A sizeable proportion of our everyday actions is driven by approach (e.g. reaching for a tasty biscuit) and avoidance (e.g. running away from a big spider) tendencies. Such tendencies can be considered fundamental motivational dimensions that steer (not only) human behaviour (Davidson & Hugdahl, 1995). These two dimensions are at the core of the framework of behavioural inhibition and activation systems (BIS and BAS, respectively; Gray, 1981; Gray & McNaughton, 1992) and can, for example, be assessed by means of the self‐report BIS/BAS questionnaire (Carver & White, 1994). Literature on individual differences in terms of inhibition and activation systems is broad and mostly focuses on disorders such as depression, anxiety, substance addictions, or obesity (Dietrich, Federbusch, Grellmann, Villringer, & Horstmann, 2014; Johnson, Turner, & Iwata, 2003; Morgan et al., 2009). There is experimental evidence that both substance addictions and obesity are related to increased approach behaviour towards problematic stimuli: While substance abuse relates to approach towards cigarettes, marijuana, or alcohol substances, obesity relates to approach tendencies towards unhealthy food cues (Cousijn et al., 2012; Mehl, Morys, Villringer, & Horstmann, 2019; Mehl, Mueller‐Wieland, Mathar, & Horstmann, 2018; Wiers et al., 2013; Wiers et al., 2014). Furthermore, obesity and higher body mass index (BMI) were shown to relate to BIS/BAS scores in a gender‐dependent fashion, with positive correlations in women, and negative correlations in men (Dietrich et al., 2014).

Regarding the neural correlates of approach/avoidance behaviours, literature suggests differential engagement of left and right frontal brain areas, such as the Brodmann area 9 or 10, and reward‐related regions of the brain, such as the nucleus accumbens or the ventral tegmental area (Aberg, Doell, & Schwartz, 2015; Tomer et al., 2013). The left hemisphere is more strongly engaged in approach, while the right one in avoidance behaviours (Aberg et al., 2015; Davidson, 1993, 1994; Sutton & Davidson, 1997; Tomer, Goldstein, Wang, Wong, & Volkow, 2008). A seminal study showed that higher alpha power, which is believed to represent inhibitory control (Bazanova & Vernon, 2014; Klimesch, Sauseng, & Hanslmayr, 2007), in right frontal brain areas (relative to the left) measured in resting‐state EEG (rsEEG), was associated with increased approach behaviour (Sutton & Davidson, 1997). This was explained by downregulated right hemispheric activity since alpha power has previously been linked to cortical inhibition by top‐down control and suppression of task‐irrelevant brain regions (Bazanova, 2012; Klimesch et al., 2007). A number of studies showed similar functional asymmetries in reward regions such as the ventral tegmental area and nucleus accumbens using positron emission tomography (Tomer et al., 2013) and task‐based fMRI (Aberg et al., 2015) during reward and punishment learning. These findings suggest that hemispheric asymmetries and their relationship to approach/avoidance behaviours can be quantified using a range of neuroimaging tools. However, the relationship between approach/avoidance behaviours and hemispheric asymmetries in resting‐state fMRI (rsfMRI) has not yet been investigated.

Since obesity is related to altered approach/avoidance behaviours, it might also be related to hemispheric asymmetries. This hypothesis is grounded in the right‐brain theory of obesity, which posits that hypoactivation of the right prefrontal cortex is an underlying factor of obesity (Alonso‐Alonso & Pascual‐Leone, 2007). It is based on findings of increased eating behaviour after damages to right‐hemispheric anterior brain areas (Regard & Landis, 1997; Short, Broderick, Patton, Arvanitakis, & Graff‐Radford, 2005). It is also supported by EEG experiments showing a higher right‐hemispheric bias for restrained eaters, a predominantly inhibitory feature (Silva, Pizzagalli, Larson, Jackson, & Davidson, 2002) and a positive relationship of left‐hemispheric bias with disinhibition and hunger (Ochner, Green, van Steenburgh, Kounios, & Lowe, 2009) as measured with the three‐factor eating questionnaire (TFEQ; Stunkard & Messick, 1985). The above‐mentioned studies, however, did not investigate a direct link between obesity measures, such as BMI and hemispheric asymmetries. Furthermore, due to the method of choice (EEG), those studies could focus mainly on cortical brain structures. Since obesity is often related to functional alterations in dopaminergic subcortical structures (Cone, Chartoff, Potter, Ebner, & Roitman, 2013; Friend et al., 2016; Geiger et al., 2009; Horstmann, Fenske, & Hankir, 2015; Narayanaswami, Thompson, Cassis, Bardo, & Dwoskin, 2013; Stice, Yokum, Burger, Epstein, & Small, 2011; Volkow, Wang, Fowler, & Telang, 2008; Vucetic, Carlin, Totoki, & Reyes, 2012), focusing on subcortical asymmetries using suitable neuroimaging techniques, such as fMRI, might further our knowledge regarding the neural correlates of obesity.

In this study, we addressed three aims using three independent samples. First, we aimed to conceptually replicate the previous findings from the literature concerning hemispheric asymmetries in terms of EEG alpha power, self‐reported approach/avoidance (BIS/BAS) and eating behaviour (TFEQ, cognitive control and disinhibition) questionnaires. This was done in a large sample of predominantly lean participants (Sample 1, 117 participants). Second, we aimed to show that the relationship of approach/avoidance, eating behaviour and rsEEG asymmetry can be extended to rsfMRI in the same sample. Here, we also aimed to investigate hemispheric asymmetries in subcortical structures, which cannot be easily done using EEG. Third, we aimed to establish the existence of obesity‐related hemispheric asymmetries in rsfMRI by investigating self‐reported eating behaviours (TFEQ), approach/avoidance behaviours (BIS/BAS), and BMI in two samples including lean, overweight, and obese participants (Sample 2, 89 participants; Sample 3, 152 participants). The three samples enabled us to provide a conceptual replication of previous studies, while at the same time expanding existing knowledge to new behavioural measures and methods.

We hypothesised that higher self‐reported approach behaviour (BAS) would be related to increased left versus right hemispheric activity, whereas higher self‐reported avoidance (BIS) would be related to increased right versus left‐hemispheric activity in both rsEEG and rsfMRI. Furthermore, increased cognitive control of food intake was expected to be related to higher right versus left‐hemispheric activity, whereas higher disinhibition was expected to be related to increased left versus right hemispheric activity. Finally, we hypothesised higher BMI to be related to increased left versus right hemispheric activity. We further aimed to investigate whether approach/avoidance‐related hemispheric asymmetries can be measured using both EEG and fMRI neuroimaging, as was previously done in a different context, for example, language research (Mazza & Pagano, 2017; Powell et al., 2006).

## 2 MATERIALS AND METHODS

Analysed data were parts of different projects, all of which were conducted according to the Declaration of Helsinki and approved by local Ethics Committees (University of Leipzig, Germany—Sample 1 and 2; Montclair State University and Nathan Kline Institute—Sample 3). All participants gave their written informed consent prior to participation.

### 2.1 Participants

#### 2.1.1 Sample 1

Sample 1 consisted of 117 healthy, right‐handed, predominantly lean participants aged 20–35 years (mean age: 25 years, mean BMI: 23.01 kg/m^2^, range: 17.95–37.80 kg/m^2^; 42 women, Table S1) taken from the “Leipzig Study for Mind‐Body‐Emotion Interactions” (Babayan et al., 2019). Exclusion criteria included: History of psychiatric or neurological disease, substance abuse, hypertension, MRI‐related contraindications (cf. table 1 in Babayan et al. (2019)). Data available for this sample included self‐reported eating (TFEQ) and approach/avoidance behaviour (BIS/BAS) questionnaires, anthropometric data (BMI), rsEEG and rsfMRI (Table S2). For analysis of EEG data, one participant was excluded due to an unresponsive electrode of interest, which resulted in a sample of 116 participants. For analysis of fMRI data, three participants were excluded due to data pre‐processing problems (failed registration), and three additional participants were excluded due to excessive head motion during data acquisition (criterion: Maximum framewise displacement exceeding 2.3 mm; Power, Barnes, Snyder, Schlaggar, & Petersen, 2012), which resulted in a sample of 111 participants.

#### 2.1.2 Sample 2

Sample 2 consisted of 89 healthy, right‐handed, lean, overweight and obese participants aged 20–37 years (mean age: 27 years, mean BMI: 29.54 kg/m^2^, range: 17.67–59.78 kg/m^2^; 73 women, Table S1). The data were collected at the Max Planck Institute for Human Cognitive and Brain Sciences in Leipzig. This sample was created by merging data of two different studies from our lab investigating decision‐making in obesity. Subsample 1 consisted of 56 lean, overweight and obese women, whereas Subsample 2 consisted of 33 participants with obesity, men and women (Mehl et al., 2019). Data available for both subsamples were self‐reported eating (TFEQ) and approach/avoidance behaviour (BIS/BAS) questionnaires, anthropometric data (BMI) and rsfMRI data (Table S2). Exclusion criteria were history of psychiatric or neurological disease, substance abuse, hypertension and MRI‐related contraindications. No participants had to be excluded during data analysis.

#### 2.1.3 Sample 3

Sample 3 consisted of participants from an open database of the enhanced Nathan Kline Institute‐Rockland Sample (NKI; http://fcon_1000.projects.nitrc.org/indi/enhanced/; releases up to 6th). From this database, we selected rsfMRI data of 152 healthy, right‐handed lean, overweight and obese participants aged 18–35 years (mean age: 24 years, mean BMI: 26.40 kg/m^2^, range: 16.26–49.96 kg/m^2^; 84 women, Table S1) with Beck Depression Inventory scores below 18 indicating lack of depressive symptoms (Beck, Steer, Ball, & Ranieri, 1996). Additional data available for this sample were self‐reported eating behaviour (TFEQ) data and anthropometric data (BMI; Table S2).

### 2.2 Questionnaire data

To investigate how hemispheric asymmetries reflect approach and avoidance behaviours, we used the BIS/BAS (behavioural inhibition system/behavioural activation system) questionnaire (Carver & White, 1994). This questionnaire was administered on Samples 1 and 2. It consists of five different scales in a revised version: Three subscales reflecting BAS (drive, reward responsivity and fun‐seeking) and two subscales reflecting BIS (anxiety and fight/flight/freeze system: Fear—FFFS fear; Heym, Ferguson, & Lawrence, 2008). According to Carver and White, the drive scale reflects persistent pursuit of desired goals; the reward responsivity scale focuses on positive responses to rewarding events; the fun‐seeking scale reflects a desire for new rewards and the inclination to approach a rewarding event. The BIS anxiety scale describes conflict detection, risk assessment and appraisal system which inhibits behaviours, while the FFFS fear scale mediates responses to aversive stimuli (Heym et al., 2008).

With regard to the self‐reported eating behaviour, we used the three‐factor eating questionnaire (TFEQ; Stunkard & Messick, 1985). It describes eating behaviour on three dimensions: Cognitive control for food (CC), disinhibition (DI) and susceptibility to hunger (H). In this study, we were predominantly interested in the first two factors, as they might reflect avoidance and approach behaviour towards food, respectively.

Within sample correlations between questionnaires measures, age and BMI can be found in Figures S1–S3.

### 2.3 Neuroimaging data

#### 2.3.1 EEG data acquisition—Sample 1

In this study, participants completed three assessment sessions in 3 days (Babayan et al., 2019). The first assessment day included a cognitive test battery and a set of questionnaires. On the second assessment day, rsEEG data were acquired, which consisted of 16 blocks, each lasting 1 min of intermittent eyes closed (EC) and eyes open (EO) conditions, summing up to a total duration of 8 min per condition. RsEEG was recorded in an acoustically shielded room with 62 active electrodes (Brain Vision ActiCAP; Brain Products GmbH, Munich, Germany) placed according to the international standard 10–20 extended localization system, also known as 10–10 extended localisation system (Oostenveld & Praamstra, 2001), all referenced to FCz electrode, with the ground electrode placed on the sternum. Electrooculographic (EOG) activity was recorded with one electrode placed below the right eye. EEG signals were sampled at 2,500 Hz and band‐pass filtered between 0.015 Hz and 1 kHz, the amplifier was set to 0.1 μV amplitude resolution and electrode impedance was kept below 5 kΩ.

#### 2.3.2 fMRI data acquisition—Sample 1

For Sample 1, MRI data were collected with a 3T Siemens Verio scanner (Siemens, Erlangen, Germany). We analysed T2*‐weighted rsfMRI, MP2RAGE and fieldmap data. RsfMRI data parameters: 657 volumes, TE = 30 ms, FA = 69°, TR = 1,400 ms, 64 slices in an interleaved order, voxel size: 2.3 × 2.3 × 2.3 mm^3^, FoV: 202 mm, multiband acceleration factor: 4, acquisition time: 15 min. MP2RAGE parameters: TE = 2.92 ms, FA1 = 4°, FA2 = 5°, TR = 2,500 ms, TI1 = 700 ms, TI2 = 2,500 ms, voxel size: 1 × 1 × 1 mm^3^, FoV: 256 mm.

#### 2.3.3 fMRI data acquisition—Sample 2

MRI data for both of the subsamples of this sample were collected with a 3 T Siemens Skyra scanner. We analysed T2*‐weighted rsfMRI, MPRAGE and fieldmap data. RsfMRI parameters: 320 volumes, TE = 22 ms, FA = 90°, TR = 2,000 ms, 40 slices in an ascending order, voxel size: 3.0 × 3.0 × 2.5 mm^3^, FoV: 192 mm, acquisition time: 11 min. MPRAGE parameters: TE = 2.01 ms, FA = 9°, TR = 2,300 ms, TI = 900 ms, voxel size: 1 × 1 × 1 mm^3^, FoV: 256 mm.

#### 2.3.4 fMRI data acquisition—Sample 3

For Sample 3, MRI data were collected with a 3 T Siemens Trio scanner. We analysed T2*‐weighted rsfMRI and MPRAGE data. RsfMRI parameters (http://fcon_1000.projects.nitrc.org/indi/enhanced/mri_protocol.html): 900 volumes in an interleaved order, TE = 30 ms, FA = 60°, TR = 645 ms, 40 slices, voxel size: 3.0 × 3.0 × 2.5 mm^3^, FoV: 222 mm, multiband acceleration factor: 4, acquisition time: 10 min. MPRAGE parameters: TE = 2.52 ms, FA = 9°, TR = 2,600 ms, TI = 900 ms, voxel size: 1 × 1 × 1 mm^3^, FoV: 256 mm.

### 2.4 Data pre‐processing

#### 2.4.1 EEG data—Sample 1

EEG data were pre‐processed using EEGLAB toolbox (version 14.1.1b; Delorme & Makeig, 2004) and custom Matlab (MathWorks, Inc, Natick, MA) scripts. EEG time series were band‐pass filtered between 1 and 45 Hz (fourth‐order back and forth Butterworth filter) and downsampled to 250 Hz. EC and EO segments were extracted and concatenated which resulted in an 8‐min block per condition. Artefactual channels and time segments were removed after visual inspection. Principal component analysis (PCA) was performed to reduce data dimensionality to *N* components (*N* ≥ 30) that explained 95% of the total variance. PCA was used for the following independent component analysis (Infomax; Bell & Sejnowski, 1995) that allowed us to reject components related to eye movements, muscle activity and heartbeats. For further analyses, the pre‐processed EEG time series were transformed to the common average reference.

#### 2.4.2 fMRI data—Samples 1 and 2

fMRI data pre‐processing for Samples 1 and 2 were identical and was done within the Nipype framework (Gorgolewski et al., 2011). In short, the pre‐processing steps included discarding the first five functional volumes, motion correction (FSL MCFLIRT; Jenkinson, Bannister, Brady, & Smith, 2002), distortion correction (FSL FUGUE; Jenkinson, Beckmann, Behrens, Woolrich, & Smith, 2012), co‐registration of the temporal mean image to the individual's anatomical image (bbregister; Greve & Fischl, 2009), denoising (rapidart and aCompCor; Behzadi, Restom, Liau, & Liu, 2007), spatial normalisation to MNI 152 2 mm (Sample 1) and 3 mm (Sample 2) standard space (ANTs; Avants et al., 2011). The details of the pipeline are described in Mendes et al., 2019.

#### 2.4.3 fMRI data—Sample 3

fMRI data pre‐processing for Sample 3 data was also done within the Nipype framework (Gorgolewski et al., 2011). In short, the pre‐processing steps included discarding first five functional volumes, motion correction (FSL MCFLIRT; Jenkinson et al., 2002), denoising (rapidart and aCompCor; Behzadi et al., 2007), removal of linear and quadratic signal trends), spatial normalisation to a 3 mm standard MNI 152 space (FSL FNIRT; Jenkinson et al., 2012). The details of the pipeline are described in Liem et al., 2017. Note that the bandpass filtering described in Liem et al. was not performed for our data, since further statistical analysis of the fMRI data (fALFF) require them to be unfiltered.

### 2.5 Neuroimaging measures

#### 2.5.1 Aim 1: EEG replication analysis

In this step, we attempted to directly replicate previous findings from Sutton and Davidson (1997) showing a positive correlation of left‐hemispheric bias with BAS–BIS differential scores. Since this measure is not recommended by authors of the BIS/BAS questionnaire (Carver & White, 1994), we used it in our study only to replicate previous findings of Sutton and Davidson (1997). In this first analysis and in this analysis only, we calculated an absolute EEG asymmetry index in frontal areas by subtracting absolute alpha power (8–12 Hz) in the F3 electrode (left) from absolute alpha power in the F4 electrode (right; asymmetry index: R–L) for mean values of EO and EC conditions together.

We then wanted to extend previous findings concerning EEG hemispheric bias and approach/avoidance behaviour to eating behaviour (as measured by the TFEQ). As rsfMRI was collected with eyes open to prevent subjects from falling asleep, our main analysis focused on EEG data from the eyes open condition in order to compare it with fMRI findings. We additionally conducted EEG analyses with relative alpha power of eyes closed condition to investigate whether potential effects observed in the eyes open condition are specific to this condition or can be extended to the eyes closed condition as well.

We focused on alpha power in the broader spectrum (8–12 Hz) and in the narrower spectrum for low alpha (8–10 Hz) for our analysis. While the broader alpha frequency band (8–12 Hz) has been previously linked to cortical inhibition by top‐down control (Bazanova, 2012; Klimesch et al., 2007), low alpha power (8–10 Hz) was previously shown to reflect general attentional demands, basic alertness, vigilance and arousal (Klimesch et al., 2007; Petsche, Kaplan, von Stein, & Filz, 1997). Including both of the measures allowed us to replicate previous results obtained using broadband alpha, and confine possible mechanistic interpretations to, for example, general attentional demands (by using low alpha). For this analysis, as opposed to the direct replication described in the previous paragraph, we used relative alpha power to control for inter‐individual differences in contaminating factors like skull thickness and meninges that might affect tissue conductivity and influence electrical signal captured at the sensor level (Babiloni et al., 2011). Relative power in broadband alpha and low alpha frequency ranges were calculated by firstly taking the mean of the squared amplitude obtained after filtering the signal in the 8–12 Hz and the 8–10 Hz frequency ranges, respectively, and then dividing it by the power within the frequency range of 4–40 Hz. In line with Sutton and Davidson (1997), relative alpha power measures were calculated in the pair of frontal electrodes F4 and F3. We also used pairs of F5/F6 and F7/F8 electrodes to extend our investigations according to current trends (Harmon‐Jones & Gable, 2018). Moreover, we included a parietal pair, P4 and P3, as a control to investigate whether the observed relationship with frontal asymmetries was topographically specific.

Previous research on hemispheric asymmetries used an absolute asymmetry index (Sutton & Davidson, 1997), while in our study we calculated a relative asymmetry index using the following equation: (R − L)/(R + L). By accounting for inter‐individual differences in alpha power magnitude, these relative indices capture asymmetries better than the absolute R − L difference and increase interpretability (Hiroshige & Dorokhov, 1997; Pivik et al., 1993). After calculation of asymmetry indices, we excluded outliers from all variables of interest using the a priori defined criterion (see section 2.6). EEG analysis for different electrodes pairs included different numbers of participants due to artefactual channels or outlier exclusions that were performed separately for each variable. We used such strategy to maximise the statistical power of our analyses.

#### 2.5.2 Aim 2 + 3: Hemispheric asymmetries in fMRI

After pre‐processing (sections 2.4.2 and 2.4.3), analysis of fMRI data in all three samples was identical. To be able to conceptually compare EEG results with fMRI results, the fractional amplitude of low‐frequency fluctuations (fALFF) was used as a measure of resting‐state brain activity (Zou et al., 2008). fALFF is usually defined as the ratio of power in the frequency range of 0.01–0.1 Hz and the power within the entire detectable frequency range. However, the samples had different sampling frequencies during fMRI data collection (i.e. repetition time, TR) and thus different detectable frequency ranges. To be able to better compare results between the samples, the denominator of the fALFF ratio was fixed to 0.00–0.25 Hz, reflecting the frequency range for the sample with the highest TR. This analysis was performed in the Nipype framework using CPAC (Configurable Pipeline for the Analysis of Connectomes, version 1.0.3, https://fcp-indi.github.io/) f/ALFF function. To compare EEG and fMRI results from our original analysis, we defined a set of regions of interest (ROI) for the fMRI analysis. Based on previous literature (Giacometti, Perdue, & Diamond, 2014; Herwig, Satrapi, & Schönfeldt‐Lecuona, 2003; Towle et al., 1993), we determined 10 ROIs that corresponded to brain areas measured by the EEG analysis in frontal (F3/F4, F5/F7 and F8/F9) and parietal (P3/P4) electrodes: Brodmann areas 6, 8, 9, 10, 44, 45 and 46 reflecting frontal contributions, Brodmann area 7, postcentral gyrus and paracentral gyrus reflecting parietal contributions. These ROIs were defined using pickatlas (Maldjian, Laurienti, Kraft, & Burdette, 2003). Since fMRI allows to investigate subcortical brain areas, for which hemispheric asymmetries have been shown (Aberg et al., 2015; Mathar et al., 2017; Tomer et al., 2008), we additionally tested a hemispheric bias in the ventral tegmental area (sphere with a 6 mm radius, coordinates based on Aberg et al., 2015; Adcock, Thangavel, Whitfield‐Gabrieli, Knutson, & Gabrieli, 2006; L: *x* = −4, *y* = −15, *z* = −9; R: *x* = 5, *y* = −14, *z* = −8), and the nucleus accumbens (sphere with a 6 mm radius, coordinates based on Aberg et al. (2015) and Neto, Oliveira, Correia, and Ferreira (2008); L: *x* = −9, *y* = 9, *z* = −8; R: *x* = 9, *y* = 8, *z* = −8). For each ROI, which was defined separately for the left and for the right hemisphere (similarly to Berkman & Lieberman, 2010), we extracted mean fALFF using SPM 12 (Wellcome Department of Cognitive Neurology, London, United Kingdom). A relative asymmetry index was calculated as follows: (L − R)/(L + R). Note that this is an inverse index compared to the one we used for EEG data, since we hypothesised that measures used in EEG and fMRI analysis are inversely correlated, due to physiological the phenomena that they are thought to measure (i.e. inhibition vs. activation, respectively). This let us directly compare relationships of EEG and fMRI data with behavioural measures, which was one of the aims of the study.

### 2.6 Statistical analysis

For each of the variables of interest, outliers were excluded based on an a priori criterion: 2.2*interquartile range below or above the first or third quartile, respectively (Hoaglin & Iglewicz, 1987; Hoaglin, Iglewicz, & Tukey, 1986; Tukey, 1977). Outliers were excluded separately for each variable to maximise the power of the analyses. Sample sizes for each analysis can be found in respective tables. Furthermore, all regression *p* values were corrected for multiple comparisons using Bonferroni correction, that is, by dividing the alpha value .05 by the number of regressions performed on the same dataset. All statistical analyses were performed using R (version 3.2.3) within JupyterNotebook.

#### 2.6.1 Aim 1: EEG replication analysis

To directly replicate Sutton's and Davidson's research (1997), for each participant we calculated the differential BAS − BIS score. We then removed outliers from both measures of interest (rsEEG and questionnaire data) and correlated BAS − BIS scores with absolute alpha asymmetry indices. To analyse the data, we performed Pearson's correlation of the obtained EEG asymmetry indices (section 2.5.1) and the BAS − BIS scores. Final sample size for this analysis after outlier exclusion was 113 participants.

To investigate the relationship between approach and avoidance behaviours and hemispheric bias as a direct replication of previous studies, we performed eight separate multiple regression analyses with asymmetry indices from relative frontal alpha power (three pairs of electrodes), relative parietal alpha power (one pairs of electrodes), relative frontal low alpha power (three pairs of electrodes) and relative parietal low alpha power (one pair of electrodes) as outcome variables. This was done separately for the EO and EC conditions. Predictors included BAS fun, BAS drive and BAS reward responsivity as well as BIS anxiety, and FFFS fear scores. Note, however, that due to the relatively low BMI range in Sample 1, in the analysis of this sample BMI served as a variable of no interest. To investigate whether gender influences the relationship between questionnaire measures and hemispheric bias, we added an interaction term with gender for each of the questionnaire variables. This was done because previous findings show that approach/avoidance behaviours might be gender‐dependent (Dietrich et al., 2014). To control for age differences, we also added this information to the model as a predictor. This and all following regression analyses were calculated using permutation tests in the “lmPerm” R package (Bonferroni corrected α = .0063).

To analyse self‐reported eating behaviour, similar regression analyses were performed as described in the previous paragraph with different questionnaire variables: Cognitive control and disinhibition (TFEQ) and their interactions with gender, and BMI, and age as variables of no interest (Bonferroni corrected α = .0063).

#### 2.6.2 Aim 2: EEG‐fMRI correspondence

##### Correlations between EEG and fMRI

First, we wanted to directly investigate the relationship of EEG asymmetries (frontal and parietal) and whole‐brain fALFF asymmetries in Sample 1 to investigate the relationships between EEG and fMRI measures. Whole‐brain fALFF asymmetries were calculated by means of (a) flipping left and right hemispheres in fALFF images (left becomes right and vice versa), (b) subtracting the flipped image from the original image, (c) adding the flipped image to the original image and (d) dividing the image obtained in Step 2 by the image obtained in Step 3. This resulted in an image of voxel‐wise values corresponding to the asymmetry index (L − R)/(L + R) (on the left side of the image, and (R − L)/(R + L) index of the right side of the brain image). A significant correlation between the EEG asymmetry index as calculated in section 2.5.1 and whole‐brain fALFF asymmetries would indicate that those two measures, even though methodologically very distinct, measure similar brain processes. This analysis was performed in SPM12 using a general linear model with voxel‐wise fALFF asymmetries as an outcome variable and the EEG asymmetry index as an explanatory variable. Results were thresholded on a voxel‐level with a 0.001 threshold and corrected for multiple comparisons using the whole‐brain 0.05 FWE‐corrected threshold.

##### Relationships between fMRI hemispheric asymmetries and approach/avoidance and eating behaviours in Sample 1

To investigate relationships of fMRI hemispheric bias with approach/avoidance and eating behaviours, we first used rotated principal component analysis (PCA) on the ROI imaging data (asymmetry indices calculated for mean fALFF values per ROI). This was done to reduce the number of comparisons in further analyses (Jolliffe & Cadima, 2016). We used the varimax rotation, which drives component loadings (correlations of components and original variables) either towards zero or towards a maximum possible value, decreasing a number of components with medium loadings, which are difficult to interpret (Jolliffe, 2002; M. B. Richman, 1986; M. L. B. Richman, 1987). As a criterion for retaining components, we chose the minimum cumulative variance explained to be over 70% (Jolliffe, 2002). This resulted in six components for each of the samples.

Furthermore, to investigate relationships of fMRI hemispheric bias and approach/avoidance behaviour, we performed a similar analysis to the one using EEG data. Six rotated principal components were defined as outcome measures, and predictors included BAS fun, BAS drive, BAS reward responsivity, as well as BIS anxiety and FFFS fear scores and their interaction with gender. Additionally, we included BMI and age as variables of no interest (Bonferroni corrected α = .0084, *n* = 110).

A similar analysis was performed to investigate relationships between fMRI hemispheric bias and eating behaviour. It included similar predictors as the EEG investigation of eating behaviour—cognitive control and disinhibition and their interaction with gender. Outcome variables were six rotated principal components. We added BMI and age as variables of no interest (Bonferroni corrected α = .0084, *n* = 106).

#### 2.6.3 Aim 3: fMRI investigations in samples including participants with obesity—relationship of hemispheric bias and self‐reported behaviours

Investigations of approach/avoidance behaviours in Sample 2 were performed similarly to the ones in Sample 1. Six rotated components were defined as outcome variables, and predictors included BIS/BAS questionnaire measures, their interaction with gender, and BMI. Age was added as a regressor of no interest (Bonferroni corrected α = .0084, *n* = 85).

A similar analysis was performed to investigate associations of self‐reported eating behaviour and hemispheric asymmetries for Samples 2 and 3. Predictor variables included eating questionnaire measures and their interaction with gender, BMI, age (regressor of no interest), while outcome variables were six rotated components (Bonferroni corrected α = .0084, Sample 2 *n* = 86, Sample 3 *n* = 140).

## 3 RESULTS

### 3.1 Aim 1: EEG replication analysis—Sample 1

In this analysis, we aimed to directly replicate findings of Sutton and Davidson (1997) of increased hemispheric bias (R − L; F4 − F3 electrodes, absolute alpha power, mean values for EO and EC conditions) being related to increased BAS − BIS differential scores. We did not find a significant relationship between those variables (*r*_(113)_ = .121, *p* = .202). Partial correlation after controlling for BMI, age and gender also did not reveal a significant relationship (*r*_(113)_ = .094, *p* = .325).

**Figure 1:**
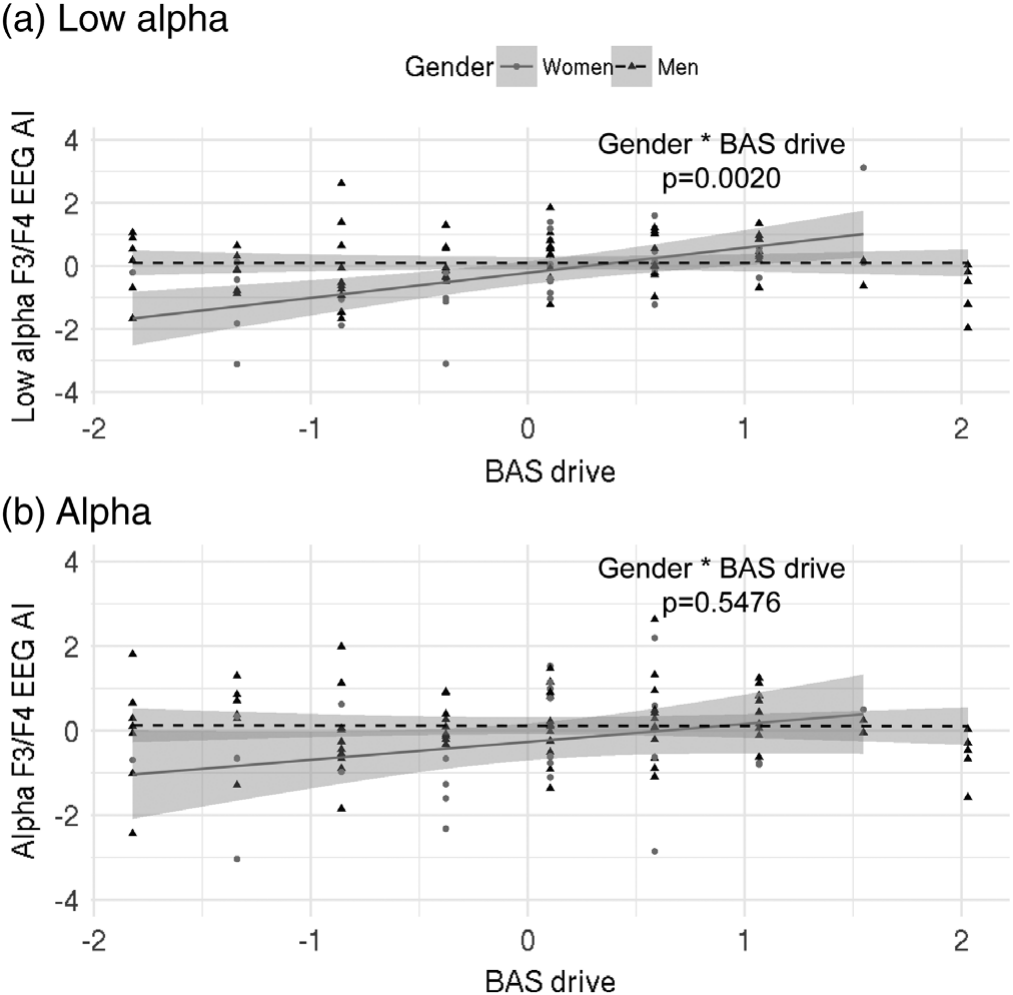
Relationship between low/full alpha EEG asymmetry index (AI) and BAS drive scores. Index used: (R − L)/(R + L). Triangles/dots represent data points, dashed/bold lines represent the best fit and grey shaded areas are 95% confidence intervals. (a) Significant correlation of hemispheric asymmetries and behavioural measures in the low alpha spectrum (beta: −.85, *p* = .0020); (b) not significant correlation of hemispheric asymmetries and behavioural measures in the broad alpha spectrum showing that the asymmetries are specific to the low alpha spectrum (beta: −.14, *p* = .5476). AI, asymmetry index; L, left; R, right

Next, we attempted to expand previous findings linking EEG and approach/avoidance behaviours to (a) additional frequency ranges to improve specificity and interpretability of findings, (b) additional questionnaire measures to improve specificity of the findings. We therefore investigated relationships between EEG parietal and frontal asymmetry indices as measured by the relative broad alpha power, as used by Sutton & Davis, and relative low alpha power. In addition to the standard broad alpha power spectrum used in previous studies, low alpha power spectrum due to its specific physiological meaning (general attentional demands, basic alertness, vigilance and arousal; Klimesch et al., 2007; Petsche et al., 1997) allowed us to more precisely interpret relationships between hemispheric asymmetries and behaviour. Here, we used the improved, relative asymmetry index: (R − L)/(R + L). For questionnaire data, we included BAS fun‐seeking, drive, reward responsivity, BIS anxiety and FFFS fear scales. First, we investigated the eyes open condition. Results of this analysis (Table 1) indicate a significant positive relationship of BAS drive and left frontal hemispheric bias in low alpha frequency for women only (BAS drive: *p* = .0009, BAS drive * gender: *p* = .0020). This is shown by an interaction of BAS drive with gender, and a significant main effect of BAS drive. In this analysis, women were coded as 0 and were the reference category, hence the main effect of BAS drive shows that this relationship is true for women, because in this case all other interaction terms including gender are also equal to zero. A similar relationship was not significant for broad alpha power. For scatter plots of these relationships see Figure 1. Even though we performed outlier exclusion prior to the analysis, we visually identified data points that could potentially be outliers and hence influence the results (points above 3 and below −3 on the *Y*‐axis, Figure 1a). Removal of these data points, however, did not alter the results. In the analysis of the eyes closed condition we found no significant effects (Table S3). We performed a linear mixed effect model analysis with subject as a random factor and condition (eyes open vs. eyes closed) as an additional fixed factor (while other questionnaire and control variables remained unchanged in the model). This was done to directly investigate whether our findings were specific to the eyes open conditions. We found a significant interaction of BAS drive, gender and condition (*p* = .0002). *Post hoc* analysis of this effect showed that the association between BAS drive and asymmetry index calculated with the relatively low alpha power is significant for women in the eyes open condition (*p* = .002). This suggests that the EEG asymmetry findings are specific to the eyes open condition only.

**Table 1:** Results of multiple regression analyses investigating the relationship between EEG asymmetry indices and approach/avoidance questionnaire measures

	Frontal												Parietal			
	Alpha F3/F4 (*n* = 104)		Low alpha F3/F4 (*n* = 107)		Alpha F5/F6 (*n* = 100)		Low alpha F5/F6 (*n* = 98)		Alpha F7/8 (*n* = 100)		Low alpha F7/F8 (*n* = 100)		Alpha (*n* = 108)		Low alpha (*n* = 109)	
	Beta	*p*‐value	Beta	*p*‐value	Beta	*p*‐value	Beta	*p*‐value	Beta	*p*‐value	Beta	*p*‐value	Beta	*p*‐value	Beta	*p*‐value
BAS fun	−.26	.3678	−.08	.7647	−.03	.8820	−.07	.7840	−.35	.0997	−.33	.1894	.04	.6863	.30	.8040
BAS fun * gender	.10	.7451	.11	.6545	.13	.5920	−.03	.9020	.52	.0307	.52	.0931	−.06	.7843	.19	.4440
BAS drive	.03	.8431	**.75**	**.0009**	.09	.4810	−.26	.1890	−.21	.3706	−.20	.2869	.36	.5158	−.27	.2610
BAS drive * gender	−.14	.5476	**−.85**	**.0020**	−.20	.4580	.19	.3040	−.04	1	.05	.7451	−.38	.4906	−.11	.8040
BAS RR	.56	.0333	.10	.6862	.29	.2330	.40	.1380	.06	.6863	.06	1.0000	−.21	.5476	.31	.3030
BAS RR * gender	−.53	.0417	−.07	.8627	−.10	.8240	−.36	.2410	.06	.8235	−.10	.8431	.18	.7059	−.30	.2600
BIS anxiety	−.14	.4155	.12	1.0000	.06	.5730	−.02	.9610	−.02	.9216	−.19	.5102	.23	.1704	.10	.9800
BIS anxiety * gender	−.02	.7647	−.36	.2228	.02	.9800	.22	.5710	−.25	.3961	.07	1.0000	−.49	.0765	.14	1.0000
FFFS fear	.46	.0240	.13	.4818	−.21	.3040	−.01	1.0000	.38	.0627	.45	.0416	.35	.0681	−.14	1.0000
FFFS fear * gender	−.39	.1766	.02	.8627	.07	.6190	−.18	.7250	−.08	.7451	−.31	.4615	−.28	.3108	.29	.1980
Age	.06	.5402	.10	.7647	−.09	.6550	−.02	1.0000	.12	.1374	.13	.1562	.20	.1647	−.34	.2000
BMI	.23	.0610	.17	.1901	−.08	.4080	−.01	1.0000	.08	.7647	.04	.9804	−.07	.3804	.00	1.0000
Gender	.47	.0230	.31	.9215	−.30	.3640	.02	.8430	.32	.0938	.28	.4184	.23	.6667	.29	.2210

*Note*: Statistically significant coefficients have been marked in bold. Note that the *p*‐value threshold after Bonferroni correction for eight separate regression analyses is .0063.

Abbreviation: RR, reward responsivity.

Furthermore, we investigated the relationship between the TFEQ and the EEG hemispheric bias. Predictor variables, in this case, included cognitive control, disinhibition, and their interactions with gender (BMI and age entered as regressors of no interest). Here, we did not find any significant associations for eyes open or eyes closed conditions. Detailed results of these analyses can be found in Tables S4 and S5.

### 3.2 Aim 2: fMRI correspondence analysis—Sample 1

First, we investigated direct relationships between EEG asymmetries (using the relative asymmetry index (R − L)/(R + L)) and whole‐brain fALFF asymmetry measures in the same sample. This analysis did not produce significant results, suggesting no correspondence between rsEEG and rsfMRI hemispheric bias measures.

Next, we investigated relationships between fMRI relative asymmetry indices (L − R)/(L + R) and approach/avoidance behaviours in Sample 1. The analysis included six retained components describing asymmetry data and questionnaire variables—BAS fun, BAS drive, BAS reward responsivity, BIS anxiety and FFFS fear and their interactions with gender. Additionally, we included BMI and age as covariates of no interest. We found no significant associations for this analysis (Tables 2 and 3, *n* = 110).

**Table 2:** Results of multiple regression analyses investigating the relationship between fMRI asymmetry indices (Sample 1) and approach/avoidance questionnaire measures

	RC1		RC2		RC3		RC5		RC4		RC6	
	Beta	*p*‐value	Beta	*p*‐value	Beta	*p*‐value	Beta	*p*‐value	Beta	*p*‐value	Beta	*p*‐value
BAS fun	.03	1.0000	.22	.5480	−.19	.9800	−.32	.9800	.02	.9800	−.09	.4110
BAS fun * gender	.08	1.0000	−.18	.5710	.34	.4900	.30	.2410	.08	.9020	−.09	.4620
BAS drive	−.20	.1870	−.24	.4380	−.23	.4300	−.09	.6600	−.06	.9220	−.08	.8240
BAS drive * gender	.13	.4620	.18	.8240	.17	.2420	.40	.2250	−.05	.9220	.11	.7840
BAS RR	.21	.2410	.01	.9800	.40	.1220	.10	.8820	−.10	.7250	−.43	.0780
BAS RR * gender	−.32	.1900	−.10	.5480	−.41	.1480	−.44	.1320	.27	.1910	.48	.0650
BIS anxiety	−.23	.3940	−.08	.6380	−.38	.0580	−.18	.6330	.20	1.0000	.17	.5410
BIS anxiety * gender	.37	.1440	.12	.4150	.46	.0350	.31	.4200	−.26	1.0000	−.28	.4320
FFFS fear	−.01	1.0000	−.03	.9410	.20	.1540	.10	.3300	.04	.4260	−.02	.8630
FFFS fear * gender	.05	1.0000	−.24	.2750	−.10	.9800	−.05	.9020	.07	.4420	.07	.7650
Age	−.05	.6060	.03	.8040	−.23	.0100	.04	.5920	−.01	.9610	.06	.5810
BMI	−.25	.0580	−.20	.0390	.03	1.0000	−.03	.7250	.07	.5710	−.11	.1810
Gender	.05	1.0000	−.38	.0220	.35	.0560	.14	.6860	.16	1.0000	−.23	.1710

*Note*: The *p*‐value threshold after Bonferroni correction for six separate regression analyses is .0084. The components have been ordered according to decreasing variance explained (Table 3). Sample size *n* = 110.

Abbreviations: RC, rotated component; RR, reward responsivity.

**Table 3:** Component loadings and cumulative variance explained for each of the rotated components (RC, Sample 1) in the BIS/BAS analysis

ROI	RC1	RC2	RC3	RC5	RC4	RC6
BA44	−0.19	0.81	0.01	0.07	0.15	0.10
BA45	0.30	0.72	−0.01	0.01	−0.22	−0.08
BA6	0.30	0.43	0.15	0.44	0.31	0.24
BA10	0.71	−0.01	0.05	−0.29	0.06	0.04
BA9	0.81	0.19	0.03	0.11	0.19	−0.02
BA8	0.82	−0.06	0.05	0.24	−0.06	−0.05
BA46	0.75	0.04	−0.3	0.01	0.05	0.09
NAcc	0.00	0.12	0.86	−0.11	0.18	0.01
VTA	0.01	0.05	−0.05	0.90	0.06	0.02
BA7	0.09	−0.05	0.08	0.12	0.85	0.07
ParacG	0.15	0.24	−0.62	−0.2	0.50	−0.08
PostcG	0.02	0.05	0.03	0.04	0.05	0.98
Cumulative variance explained	0.22	0.34	0.45	0.55	0.65	0.74

*Note*: ROIs represent 12 regions of interest selected for the fMRI analyses.

Abbreviations: BA, Brodmann area; NAcc, nucleus accumbens; ParacG, paracentral gyrus; PostcG, postcentral gyrus; ROI, region of interest; VTA, ventral tegmental area.

Furthermore, we investigated whether hemispheric asymmetries measured with fMRI are related to self‐reported eating behaviour (TFEQ). This analysis included cognitive control, disinhibition and their interactions with gender as predictor variables, while the outcome variables were the six rotated components from the PCA analysis. Variables of no interest were BMI and age. Here, we did not find any significant relationships. Results of this analysis can be found in Tables S6 and S7 (*n* = 106).

### 3.3 Aim 3: fMRI investigations in samples including participants with obesity—relationship of hemispheric bias and self‐reported behaviours

Here, we investigated relationships between fMRI relative asymmetry indices (L − R)/(L + R) and approach/avoidance behaviours in Sample 2, characterised by a wider BMI range including individuals with overweight and obesity. The analysis included the six retained components describing asymmetry data as outcome variables and questionnaire variables—BAS fun, BAS drive, BAS reward responsivity, BIS anxiety, FFFS fear, their interactions with gender and BMI as predictors. Additionally, we included age as a regressor of no interest. Sample size for this analysis was 85.

**Figure 2:**
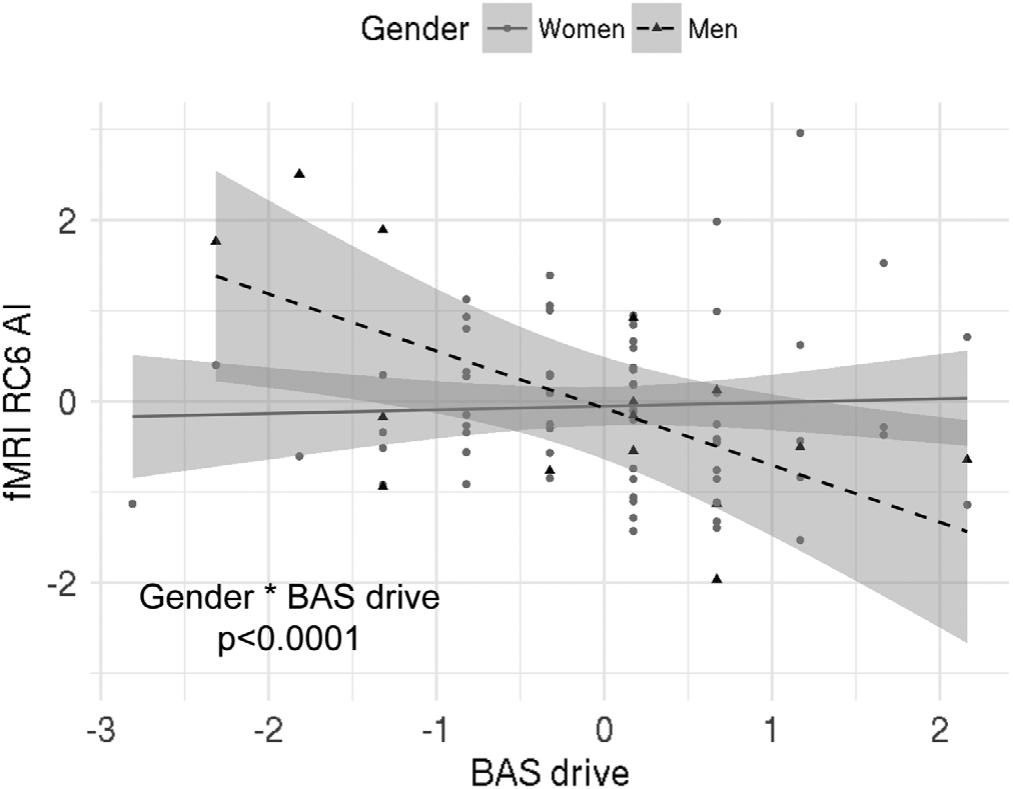
Relationship between RC6 and BAS drive scores in Sample 2; there was a significant interaction effect of BAS drive scores and gender on RC6. Index used: (L − R)/(L + R). Triangles/dots represent data points, dashed/bold lines represent the best fit and grey shaded areas are 95% confidence intervals. AI, asymmetry index; L, left; R, right; RC, rotated component

**Figure 3:**
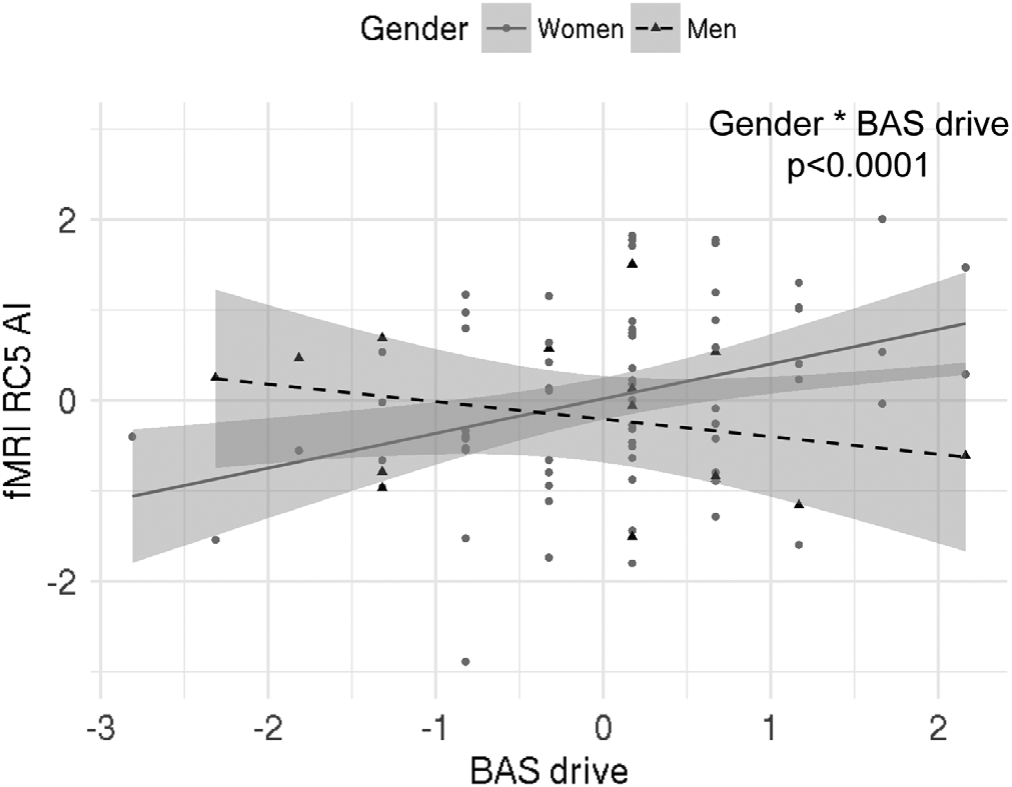
Relationship between RC5 and BAS drive scores in Sample 2; there was a significant interaction effect of BAS drive scores and gender on RC5, and a significant effect of BAS drive scores on RC5 in women. Index used: (L − R)/(L + R). Triangles/dots represent data points, dashed/bold lines represent the best fit and grey shaded areas are 95% confidence intervals. AI, asymmetry index; L, left; R, right; RC, rotated component

**Figure 4:**
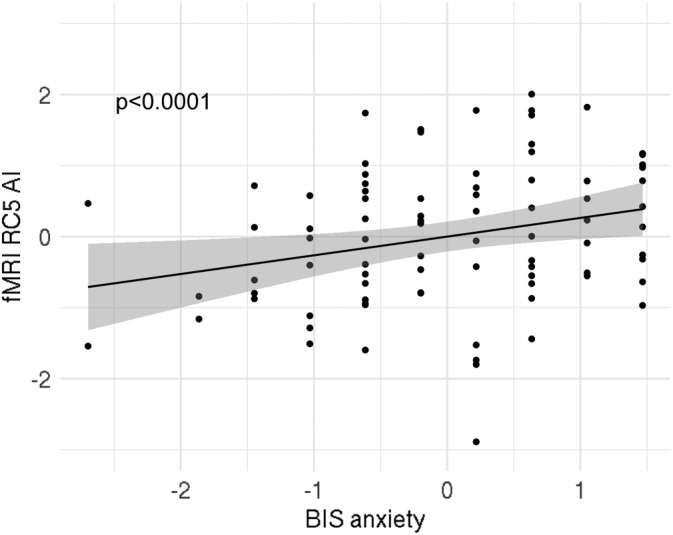
Relationship between RC5 and BIS anxiety scores in Sample 2; there was a significant interaction effect of BIS anxiety scores on RC5. Index used: (L − R)/(L + R). Dots represent data points, line represents best fit and grey shaded areas are 95% confidence intervals. AI, asymmetry index; L, left; R, right; RC, rotated component

We found a significant interaction effect of BAS Drive and gender on the rotated component 6 (RC6; Table 4). This component is strongly influenced by the BA10 (Table 5). This suggests that in men increased left over right‐brain activity in the BA10 is related to lower BAS drive scores, while in women increased left over right brain activity is related to higher BAS drive scores (Figure 2). Furthermore, the results showed a significant interaction effect of BAS drive and gender on RC5, and a main effect of BAS drive on RC5 with contributions from the VTA (Tables 4 and 5). It suggests that in women increased left over right hemispheric activity in the VTA is related to increased BAS drive scores (Figure 3). Finally, we also found a significant association between RC5 scores and BIS anxiety (Tables 4 and 5), suggesting that increased left versus right activity in the VTA is related to increased BIS anxiety scores independent of gender (Figure 4).

**Table 4:** Results of multiple regression analyses investigating the relationship between fMRI asymmetry indices (Sample 2) and approach/avoidance questionnaire measures

	RC1		RC2		RC4		RC6		RC3		RC5	
	Beta	*p*‐value	Beta	*p*‐value	Beta	*p*‐value	Beta	*p*‐value	Beta	*p*‐value	Beta	*p*‐value
BAS fun	.03	.8824	−.13	.6030	.06	.4380	−.04	.6860	−.10	.4110	−.04	.7450
BAS fun * gender	−.06	1.0000	−.43	.3110	−.40	.3180	−.19	1.0000	.28	.3210	.29	.9020
BAS drive	.00	1.0000	−.09	.3300	−.02	1.0000	.19	.2120	.00	1.0000	**.36**	**<.0001**
BAS drive * gender	−.02	.9216	−.29	.6150	.59	.5330	**−.90**	**<.0001**	.14	.6550	**−.53**	**<.0001**
BAS RR	.06	.9216	.11	.3730	.19	.8240	−.05	.9220	−.09	.9800	−.01	.9610
BAS RR * gender	.16	.6429	−.07	.6600	−.53	.0220	.38	.6670	−.20	1.0000	−.30	.5410
BIS anxiety	.29	.4444	.39	.1080	−.42	.0600	.14	1.0000	.06	.9410	**.67**	**<.0001**
BIS anxiety * gender	−1.24	.3125	−.72	.4110	1.46	.3440	−.50	.9020	1.05	.5730	−.13	.9610
FFFS fear	−0.27	.2724	−.49	.0680	.27	.2200	−.28	.1220	.20	.4730	−.34	.1490
FFFS fear * gender	1.23	.0971	1.14	.1870	−1.38	.1580	−.04	.9800	−.70	.6600	−.31	.7250
Age	.17	1.0000	.08	.4910	.19	.0610	.12	.2710	.10	.6600	.04	1.0000
BMI	.14	.5102	.04	.9610	−.04	.9020	−.17	.2930	.08	.3070	.09	.6330
Gender	.46	.6863	−.52	.5640	−.38	.7840	1.47	.7650	−.82	.6030	.93	.5330

*Note*: The *p*‐value threshold after Bonferroni correction for six separate regression analyses is .0084. The components have been ordered according to decreasing variance explained (Table 5). Sample size *n* = 85.

Abbreviations: RC, rotated component; RR, reward responsivity.

**Table 5:** Component loadings for each of the PCA's rotated components (RC, Sample 2) in the BIS/BAS analysis. ROIs represent 12 regions of interest selected for the fMRI analyses

ROI	RC1	RC2	RC4	RC6	RC3	RC5
BA44	0.73	−0.19	0.08	0.13	−0.04	0.06
BA45	0.34	0.69	−0.19	0.17	−0.04	0.05
BA6	0.18	0.06	0.10	0.32	0.73	−0.01
BA10	0.01	0.15	−0.02	0.90	0.17	−0.12
BA9	0.58	0.05	−0.21	−0.25	0.02	−0.58
BA8	0.68	0.18	0.17	−0.01	0.10	0.00
BA46	0.39	−0.49	−0.04	0.47	−0.25	0.11
NAcc	−0.08	−0.06	−0.08	−0.09	0.79	−0.02
VTA	0.10	0.09	−0.10	−0.16	−0.03	0.90
BA7	0.08	−0.10	0.85	0.07	−0.12	−0.08
ParacG	−0.17	0.73	0.23	0.05	−0.04	0.07
PostcG	0.14	0.36	0.66	−0.15	0.16	0.06
Cumulative variance explained	0.14	0.27	0.38	0.49	0.60	0.70

Abbreviations: BA, Brodmann area; VTA, ventral tegmental area; NAcc, nucleus accumbens; ParacG, paracentral gyrus; PostcG, postcentral gyrus; ROI, region of interest.

Furthermore, we investigated whether relative hemispheric asymmetries measured with fMRI (L − R)/(L + R) are related to self‐reported eating behaviour in Samples 2 and 3 (characterised by a wider BMI range). These analyses included cognitive control, disinhibition, their interactions with gender and BMI as predictor variables, while the outcome variables were six rotated components from the PCA. Age was entered as a regressor of no interest. Our analyses revealed no relationships between hemispheric asymmetries and eating behaviour in both samples. Details of these analyses can be found in Tables S8–S11. Sample sizes for these analyses were 86 and 140 for Samples 2 and 3, respectively.

## 4 DISCUSSION

In this study, we aimed at replicating previous EEG findings concerning relationships of resting‐state hemispheric asymmetries and approach/avoidance behaviours in healthy participants. Second, we aimed to investigate whether EEG asymmetry findings and fMRI asymmetry findings correspond to each other in the approach/avoidance context, as they do in the language (e.g. syntactic and semantic processing), or attention context (e.g. object or face perception; Chakrabarty et al., 2017; Mazza & Pagano, 2017; Powell et al., 2006). Importantly, we also used fMRI to obtain data from subcortical structures, which are not easily obtainable from the EEG measures. This is an important addition especially in the context of obesity, since alterations in functions and structure of subcortical dopaminergic regions were previously often related to obesity (Cone et al., 2013; Friend et al., 2016; Geiger et al., 2009; Horstmann et al., 2015; Narayanaswami et al., 2013; Stice et al., 2011; Volkow et al., 2008; Vucetic et al., 2012). Furthermore, we attempted to expand the findings to self‐reported eating behaviour and BMI (which has been related to increased approach behaviour; Mehl et al., 2019; Mehl et al., 2018) using rsfMRI. We tested three independent samples to answer these questions. In Sample 1, we were not able to directly replicate Sutton's and Davidson's EEG findings showing a positive association between BAS − BIS scores (describing individual differences between approach and avoidance behaviours) and higher left resting‐state hemispheric bias. However, we show a conceptual replication of this bias with BAS drive in women. Second, we were not able to find significant associations between rsfMRI data and approach/avoidance behaviours in the same Sample. Furthermore, in Sample 2—which included participants with overweight and obesity as well as rsfMRI data—we found significant associations between hemispheric asymmetries, gender, BAS drive and BIS anxiety. Finally, in none of the samples did we find significant relationships of hemispheric bias and self‐reported eating behaviour or BMI.

Past work by Grey and colleagues has suggested that human behaviour is driven by the interplay of the behavioural inhibition and activation systems (Gray, 1981; Gray & McNaughton, 1992). In a number of clinical and laboratory studies, it has been proposed that those fundamental behavioural dimensions are driven by asymmetric engagements of anterior brain regions (Davidson & Hugdahl, 1995; Harmon‐Jones & Gable, 2018). In particular, the neural substrate for the inhibition system or withdrawal behaviour was found in the right prefrontal cortex, while the left prefrontal cortex was related to approach behaviour (Davidson & Hugdahl, 1995; Harmon‐Jones & Gable, 2018). Those conclusions are based predominantly on rsEEG studies but also on studies in patients with frontal brain lesions. In our work, we aimed to replicate the seminal study by Sutton and Davidson (1997), which showed a positive association of BAS − BIS differential scores with left hemispheric bias, as measured by absolute alpha power from rsEEG. Although we have analysed our data in the same way, we did not replicate these results. In our study, the rsEEG duration was 16 min (eyes closed + eyes open) as opposed to 8 min in Sutton's study (eyes closed + eyes open), however, longer duration might provide a better estimation of resting‐state processes. Yet it is unlikely that those small methodological differences can explain the lack of direct replication. However, our sample size was much larger and included participants in a wider age‐range (20–35 years). Additionally, gender distribution was not equal, whereas in Sutton's study 50% of the sample were women (although we statistically controlled for age, BMI and gender). Those factors might influence results beyond what is possible to be corrected by means of statistical analysis.

Importantly, in a more detailed EEG data analysis using a refined relative asymmetry index, that is superior to an absolute in terms of interpretability, and relative alpha power, we found effects that are conceptually similar to the ones by Sutton and Davidson (1997): We found a positive gender‐specific relationship between left hemispheric bias (indicating increased left over right hemispheric activity) and BAS drive. Additional analyses showed that this effect is specific for the eyes open condition, as in the eyes closed condition we did not find any significant associations between hemispheric asymmetries and approach/avoidance behaviours. It is conceivable that BIS/BAS correlates only with EEG hemispheric asymmetries during an EO resting condition because approach/avoidance behaviours require engaging with the environment in order to perceive and react to stimuli.

The effect observed in EEG analysis in eyes open condition indicates that higher approach behaviour (or drive towards positive reinforcement) is related to higher left‐brain activity at rest. While Sutton and Davidson (1997) found a similar association in a sample including both genders, in our sample, it was only true for women. As Sutton and Davidson did not explicitly test gender differences, it cannot be excluded that their findings were driven by women. Furthermore, in this study, we found significant effects using a different measure of approach behaviour (BAS drive vs. BAS − BIS score). BAS drive describes an absolute strength of the approach system (drive towards positive stimuli). BAS − BIS difference score is conceptually and psychometrically inappropriate (Carver & White, 1994), but we used it nevertheless only to directly replicate findings of Sutton and Davidson (1997). It is possible that those different measures are related to hemispheric asymmetries in a distinct, gender‐dependent way. Additionally, previous literature shows that gender indeed might influence hemispheric asymmetries—brains of men seem to be more lateralised as compared to women (Hausmann, 2002, 2017; McGlone, 1980). This does not exclude the possibility that women's brains show different associations between hemispheric asymmetries and self‐reported behaviours, possibly through sex hormones (Hausmann, 2002, 2017). Future studies should aim to replicate our result and investigate asymmetries specifically with regard to gender differences.

It is worth noting that we found significant associations of questionnaire measures and hemispheric asymmetries measured with low relative alpha power, but not with broadband relative alpha power. Since low alpha power represents such attentional processes as vigilance (Klimesch et al., 2007; Petsche et al., 1997), our results suggest that hemispheric asymmetries are related to those processes, rather than to general inhibitory processing within the brain.

The second aim of our study was to investigate whether approach/avoidance‐related asymmetries can be measured with both EEG and fMRI. We were not able to replicate EEG findings in Sample 1 using rsfMRI. Such lack of replication might be related to the fact that alpha power and fALFF measure different processes. This is also reflected in a lack of direct relationship between EEG and whole‐brain fALFF asymmetries. Alpha power is indeed conceptualised to be inversely related to brain activity by enabling active inhibition (Klimesch et al., 2007). fALFF, on the other hand, is generally suggested to be a measure of brain activity (Zou et al., 2008). For example, low‐frequency fluctuations (LFFs) in grey matter were previously found to be higher than in white matter suggesting that they reflect grey matter metabolism and activity (Biswal, Yetkin, Haughton, & Hyde, 1995). This claim was further substantiated by a study which created a map of resting fluctuations in the visual cortex, suggesting that LFFs reflect spontaneous brain activity (Kiviniemi et al., 2000). Spontaneous LFFs were also identified in the default mode network at rest, again, suggesting that they might reflect brain activity (Fransson, 2005). We therefore hypothesised that alpha power and fALFF could simply be inversely related to each other. This is, however, not supported by our data. Instead, this relationship seems to be more complex. This might be because EEG and fMRI measure predominantly post‐synaptic potentials and BOLD response, respectively (Bucci & Galderisi, 2011; Gauthier & Fan, 2019). The physiological basis of the two are hugely different. Post‐synaptic potentials measured by EEG are a direct reflection of neuronal activity, while BOLD response is an indirect measurement of neuronal activity through quantification of oxygen consumption of neurons. Additionally, EEG and fMRI measure oscillations within very different frequency ranges (8–12 Hz vs. 0.01–0.1 Hz, respectively).

Interestingly, in Sample 2, which included overweight and obese individuals we replicated EEG findings from Sample 1: We found relationships between rsfMRI and BAS drive questionnaire. Here, women showed a positive relationship between BAS drive and left vs. right hemispheric activity in the rotated components highly related to the BA10 and VTA. Additionally, we found a significant positive association between BIS anxiety and left versus right hemispheric activity in the component related to the VTA. Findings of the VTA in the context of approach/avoidance behaviour and hemispheric asymmetry are novel, because previous studies used predominantly EEG to measure brain activity, which makes it difficult to obtain measures of activity from subcortical brain regions. The association of VTA and BAS drive confirms our hypothesis that the left brain hemisphere is predominantly related to approach behaviour. However, the association between VTA and BIS anxiety points to an opposite pattern (Harmon‐Jones & Gable, 2018). Here, we show that the relationship between hemispheric asymmetries, as measured by fMRI and fALFF, and BAS drive, is similar to the one found in the EEG data. This is interesting for two reasons: First, it suggests that there might be an indirect relationship between two fundamentally different (Scheeringa et al., 2011) measures of brain activity (by means of correlations with the same behavioural measures). Second, it shows that fMRI measures of hemispheric asymmetry can be related to approach and avoidance behaviours. This provides additional methodological possibilities to investigate relationships between hemispheric asymmetries and behavioural measures of approach/avoidance. However, it is important to keep in mind the limitation that we were not able to replicate EEG results in the same sample using fMRI. Thus, we conclude that the measure of hemispheric asymmetries utilising fALFF and the relationship of this measure with approach/avoidance behaviour seem to be unstable and possibly dependent on the characteristics of samples under study, predominantly the BMI distribution. More research is needed to investigate which different measures influence this relationship. One way to improve current research is to use large and well‐characterised publicly available datasets.

We further investigated the relationship between hemispheric bias and BMI, since BMI in the obese range is related to increased approach behaviour (Mehl et al., 2018; Mehl et al., 2019) and obesity has been described as a deficiency of right‐brain activation (Alonso‐Alonso & Pascual‐Leone, 2007). This was done in Samples 2 and 3, since they included participants with BMI in the overweight and obese range. Our analyses did not show a significant relationship between hemispheric bias and BMI. Thus, we did not find support for the right‐brain theory of obesity, which suggests that hemispheric biases at rest may not be related to BMI per se, but to specific patterns of approach/avoidance and/or eating behaviour instead. Relatedly, it is conceivable that hemispheric biases during specific task performance might be related to BMI. While previous studies supporting the right brain theory of obesity largely focused on patients with unilateral brain lesions or structural asymmetries (Colcombe et al., 2006; Regard & Landis, 1997; Short et al., 2005; Uher & Treasure, 2005), our resting‐state data were obtained in neurologically healthy participants. This may imply that previous results on obesity‐related hemispheric asymmetries cannot be generalised to individuals with obesity. This heterogeneity, while increasing ecological validity, might introduce noise, which in turn makes it difficult to detect associations between BMI and hemispheric asymmetries. Finally, the right‐brain theory of obesity is based on a number of findings relating eating behaviours and physical activity to hemispheric asymmetries, and not to BMI directly (Colcombe et al., 2006; Regard & Landis, 1997; Short et al., 2005; Uher & Treasure, 2005), as did our study—which might explain deviating results. In sum, future studies need to focus on relationships between obesity measures and hemispheric asymmetries in EEG and fMRI measurements of both resting‐state and task contexts to confirm or revise the right‐brain theory of obesity.

Finally, we investigated associations between hemispheric asymmetries and self‐reported eating behaviours in all three samples. Here, we did not find any relationships using rsEEG and rsfMRI data. That is, we were not able to replicate previous rsEEG findings showing hemispheric bias relationships with disinhibition, hunger (Ochner et al., 2009) or restrained eating (Silva et al., 2002). Similarly, the study by Ochner et al. (2009) included participants with overweight and obesity (so did 2 of our 3 samples), and the study by Silva et al. (2002) included only lean women (one of our samples included mostly lean participants and we investigated interactions with gender). However, certain differences between those studies and our research exist, which might explain different results: First, Ochner and colleagues investigated a group of much older participants (mean age: 49 years). It is conceivable that the duration of obesity influences prefrontal asymmetries, hence age might explain differences between results. Furthermore, in our study, we were very conservative with regard to multiple comparisons correction, while Ochner and colleagues were more liberal in this respect.

Some limitations of our study need to be acknowledged: EEG data were only available for one sample. It would provide additional evidence to investigate differences between rsEEG and rsfMRI asymmetry associations with behavioural measures in other samples, especially concerning BMI and eating behaviour—aspects not investigated as thoroughly as approach/avoidance behaviours. As our study investigated relationships between self‐reported approach/avoidance behaviours and resting‐state neuroimaging measures, future studies could also include task‐based neuroimaging measures, especially in the context of obesity. This might give a more valid proxy for everyday motivational behaviours and therefore have higher ecological validity.

In sum, we conceptually replicated findings showing relationships between hemispheric bias and approach/avoidance behaviours in women, but not self‐reported eating behaviour in both rsEEG and rsfMRI. Moreover, we investigated relationships between rsEEG alpha power measures and rsfMRI fALFF. We show that associations of hemispheric asymmetries measured with rsEEG and rsfMRI are similar, however, we do not provide a replication of rsEEG results and rsfMRI results in the same sample. Future studies should answer the question of how those measures relate to each other in a more systematic way. We suggest that future studies should be performed using samples of lean, overweight and obese participants using both EEG and fMRI measures.

## CONFLICT OF INTEREST

All authors declare no conflict of interest.

## Supporting information


**Appendix S1**: Supporting information

## Data Availability

The data that support the findings of this study are available from the corresponding author upon reasonable request.
