# Supplementary material for: Hemispheric asymmetries in resting‐state EEG and fMRI are related to approach and avoidance behaviour, but not to eating behaviour or BMI
Source: Hum Brain Mapp. 2019 Nov 21;41(5):1136–52. doi: 10.1002/hbm.24864 (PMC7267939; doi:10.1002/hbm.24864)

**Title**

Hemispheric asymmetries in resting-state EEG and fMRI are related to approach and avoidance behaviour, but not to eating behaviour and BMI

**Authors**

Filip Morys^1, 2, 3^, Lieneke Janssen^1, 2^, Elena Cesnaite^2^, Frauke Beyer^2, 4^, Isabel Garcia-Garcia^2,^ ^3^, Jana Kube^2, 5^, Deniz Kumral^2, 6^, Franziskus Liem^7, 8^, Nora Mehl^2, 9^, Keyvan Mahjoory^2, 10^, Anne Schrimpf^2^, Michael Gaebler^2, 6^, Daniel Margulies^2, 8, 11^, Arno Villringer^2, 6^, Jane Neumann^1, 2, 12^, Vadim Nikulin^2, 13, 14^, Annette Horstmann^1, 2, 4, 15^

**Affiliations**

^1^ Leipzig University Medical Centre, IFB Adiposity Diseases, 04103 Leipzig, Germany

^2^ Department of Neurology, Max Planck Institute for Human Cognitive and Brain Sciences, 04103 Leipzig, Germany

^3^ Montreal Neurological Institute, McGill University, Montreal, QC, Canada

^4^ Subproject A5, CRC1052 “Obesity Mechanisms”, University of Leipzig, Leipzig, Germany

^5^ Brandenburg University of Technology Cottbus-Senftenberg, 03013 Cottbus, Germany

^6^ MindBrainBody Institute at the Berlin School of Mind and Brain, Humboldt-Universitaet zu Berlin, Berlin, Germany

^7^ University Research Priority Program “Dynamics of Healthy Aging”, University of Zurich, CH-8006 Zurich, Switzerland

^8^ Max Planck Research Group for Neuroanatomy & Connectivity, Max Planck Institute for Human Cognitive and Brain Sciences, Leipzig, Germany

^8^ Faculty of Psychology, Technical University Dresden, 01062 Dresden, Germany

^10^ University of Muenster, Institute for Biomagnetism and Biosignal Analysis, 48149 Muenster, Germany

^11^ Brain and Spine Institute, 75013 Paris, France

^12^ Ernst-Abbe-Hochschule – University of Applied Sciences, 07745 Jena, Germany

^13^ Centre for Cognition and Decision Making, Institute for Cognitive Neuroscience, National Research University Higher School of Economics, Moscow, Russia

^14^ Department of Neurology, Charité – Medical University Berlin, 10117 Berlin, Germany

^15^ Department of Psychology and Logopedics, Faculty of Medicine, University of Helsinki

# Supplementary results

## Samples comparison

To compare the three samples regarding their demographic characteristics we ran separate one-way ANOVAs for BMI and age, and a χ^2^ test for equality of gender distribution between the samples. We followed up the ANOVAs with *post hoc* Tukey’s tests to determine which groups differed from each other. The results of these analyses can be found in Table S1. We found that regarding BMI all samples differed from each other, while regarding age, Sample 2 differed from both other samples, which did not significantly differ from each other. Concerning gender distribution, all samples differed from each other.

## Questionnaire data – samples comparison and correlations

Table S1 represents questionnaire data of all 3 samples included in the study. Performed ANOVAs indicated group differences in both cognitive control and disinhibition scales. Tukey’s tests showed that concerning cognitive control there were no significant pairwise differences between groups. Regarding disinhibition, Sample 2 differed significantly from both Samples 1 and 3. We further performed four ANOVAs to compare BIS/BAS data in Samples 1 and 2. This analysis revealed no significant differences for this questionnaire. Within sample correlations between questionnaire measures, age, and BMI can be found in supplementary Figures S1-S3.

# Supplementary Tables

Table S1 Means, standard deviation and statistical tests concerning questionnaire differences in all experimental samples. BIS/BAS questionnaire data were available for Samples 1 and 2, whereas TFEQ data were available for all samples. CC – cognitive control, DI – disinhibition, BAS – behavioural activation system, BIS – behavioural inhibition system.

|  | Sample 1 (n=117) | | | Sample 2 (n=89) | | | Sample 3 (n=152) | | | ANOVA | | Sample 1 vs. Sample 2 | Sample 1 vs. Sample 3 | Sample 2 vs. Sample 3 |
| --- | --- | --- | --- | --- | --- | --- | --- | --- | --- | --- | --- | --- | --- | --- |
|  | Mean | SD | Range | Mean | SD | Range | Mean | SD | Range | F(2,355)-value | p-value |  |  |  |
| BMI (kg/m^2^) | 23.01 | 2.57 | 17.95-31.80 | 29.54 | 8.25 | 17.67-59.78 | 26.40 | 5.62 | 16.26-49.96 | 33.70 | **<0.0001** | **<0.0001** | **<0.0001** | **<0.0001** |
| Age (years) | 25 | 3 | 20-35 | 27 | 4 | 20-37 | 24 | 4.5 | 18-35 | 18.08 | **<0.0001** | **<0.0001** | **<0.0001** | **<0.0001** |
| CC | 5.56 | 4.53 | 0-18 | 6.92 | 4.60 | 0-20 | 6.78 | 4.57 | 0-20 | 3.073 | **0.0480** | 0.0885 | 0.0777 | 0.9719 |
| DI | 5.23 | 2.48 | 1-12 | 6.79 | 3.52 | 1-15 | 4.63 | 2.90 | 1-15 | 15.36 | **<0.0001** | **0.0006** | 0.2158 | **<0.0001** |
|  | | | | | | | | | | F(1,204) | p-value |  | | |
| BAS fun | 3.13 | 0.41 | 2.25-4.00 | 3.03 | 0.50 | 1.75-4.00 | - | - |  | 2.213 | 0.1380 | - | - | - |
| BAS drive | 2.94 | 0.51 | 2.00-4.00 | 2.92 | 0.51 | 1.50-4.00 | - | - |  | 0.103 | 0.7490 | - | - | - |
| BAS reward responsivity | 3.41 | 0.38 | 2.40-4.00 | 3.33 | 0.34 | 2.60-4.00 | - | - |  | 2.561 | 0.1110 | - | - | - |
| BIS anxiety | 3.08 | 0.53 | 1.50-4.00 | 3.103 | 0.53 | 1.50-4.00 | - | - |  | 0.075 | 0.7840 | - | - | - |
| FFFS fear | 2.72 | 0.56 | 1.33-4.00 | 2.67 | 0.54 | 1.00-3.00 | - | - |  | 0.280 | 0.5980 |  |  |  |
| Gender |  | | | | | | | |  | χ^2^ | |  | | |
|  |  |  |  |  |  |  |  |  |  | test value | p-value |  |  |  |
|  | 42 women | |  | 73 women | |  | 84 women | |  | 53.636 | **<0.0001** | **<0.0001** | **0.0001** | **<0.0001** |

Table S2 Available data for each of the investigated samples. x marks available datasets.

| Data | Sample 1 (n=117) | Sample 2 (n=89) | Sample 3 (n=152) |
| --- | --- | --- | --- |
| TFEQ | x | x | x |
| BIS/BAS | x | x |  |
| Anthropometric data (BMI) | x | x | x |
| rsEEG | x |  |  |
| rsfMRI | x | x | x |

TFEQ – three factor eating questionnaire; BIS/BAS – behavioural activation/inhibition system questionnaire; rsEEG – resting-state EEG; rsfMRI – resting-state fMRI

Table S3 Table with results of multiple regression analyses investigating the relationship between EEG asymmetry indices and approach/avoidance questionnaire measures for the eyes closed condition. Statistically significant coefficients have been marked in bold. Note that the p-value threshold after Bonferroni correction for 8 separate regression analyses is 0.0063. RC – rotated component, RR – reward responsivity

|  | Frontal | | | | | | | | | | | | Parietal | | | |
| --- | --- | --- | --- | --- | --- | --- | --- | --- | --- | --- | --- | --- | --- | --- | --- | --- |
|  | Alpha F4/F4 (n=108) | | Low alpha F3/F4 (n=108) | | Alpha F5/F6 (n=105) | | Low alpha F5/F6 (n=105) | | Alpha F7/8 (n=97) | | Low alpha F7/F8  (n=98) | | Alpha (n=108) | | Low alpha (n=108) | |
|  | Beta | p-value | Beta | p-value | Beta | p-value | Beta | p-value | Beta | p-value | Beta | p-value | Beta | p-value | Beta | p-value |
| BAS fun | -0.36 | 0.1603 | -0.48 | 0.0456 | 0.33 | 0.2890 | 0.58 | 0.0397 | -0.08 | 1.0000 | 0.00 | 1.0000 | 0.38 | 0.0988 | 0.41 | 0.0547 |
| BAS fun * gender | 0.16 | 0.4082 | 0.38 | 0.1349 | -0.13 | 0.4770 | -0.49 | 0.0597 | 0.11 | 1.0000 | -0.05 | 0.863 | -0.33 | 1.0000 | -0.27 | 0.2776 |
| BAS drive | 0.08 | 1.0000 | 0.04 | 1.0000 | 0.04 | 1.0000 | -0.04 | 1.0000 | 0.14 | 0.5714 | -0.16 | 0.745 | 0.15 | 0.4296 | -0.27 | 0.8431 |
| BAS drive * gender | -0.15 | 0.9804 | -0.08 | 1.0000 | 0.14 | 0.9610 | 0.27 | 0.6429 | -0.21 | 0.5 | 0.23 | 0.633 | -0.09 | 0.5942 | 0.46 | 0.0816 |
| BAS RR | 0.34 | 0.1554 | 0.35 | 0.1711 | -0.23 | 0.3330 | -0.02 | 0.9608 | -0.07 | 0.5942 | 0.07 | 0.804 | -0.18 | 0.3818 | -0.18 | 0.8824 |
| BAS RR * gender | -0.35 | 0.1822 | -0.45 | 0.1687 | 0.08 | 0.7250 | -0.17 | 0.5102 | -0.22 | 0.5811 | -0.39 | 0.162 | 0.17 | 0.6863 | 0.00 | 1.0000 |
| BIS anxiety | -0.49 | 0.0581 | 0.23 | 0.5275 | 0.32 | 0.2910 | 0.21 | 0.1924 | 0.22 | 0.5102 | 0.14 | 0.377 | 0.04 | 0.8235 | 0.15 | 0.33 |
| BIS anxiety * gender | 0.46 | 0.0933 | -0.32 | 0.4904 | -0.29 | 0.2910 | -0.15 | 0.3285 | -0.21 | 0.4737 | -0.17 | 0.375 | 0.03 | 0.9608 | 0.07 | 0.9608 |
| FFFS fear | -0.17 | 0.3317 | -0.38 | 0.1392 | 0.29 | 0.4320 | 0.14 | 1.0000 | 0.43 | 0.0306 | 0.34 | 0.11 | 0.29 | 0.1512 | 0.21 | 0.7647 |
| FFFS fear * gender | 0.19 | 0.4082 | 0.37 | 0.3529 | -0.37 | 0.4010 | -0.10 | 1.0000 | -0.40 | 0.1055 | -0.24 | 0.686 | -0.39 | 0.1826 | -0.30 | 0.8627 |
| Age | -0.05 | 0.4027 | -0.08 | 0.2203 | 0.04 | 0.8430 | 0.00 | 1.0000 | -0.01 | 1.0000 | 0.15 | 0.101 | -0.09 | 1.0000 | -0.02 | 0.6545 |
| BMI | 0.09 | 0.9412 | 0.19 | 0.0251 | -0.05 | 0.5220 | -0.05 | 0.6154 | 0.07 | 0.5158 | 0.05 | 0.843 | -0.13 | 0.3122 | -0.09 | 0.5213 |
| Gender | 0.22 | 0.1657 | 0.30 | 0.8824 | -0.01 | 1.0000 | 0.02 | 0.6863 | 0.17 | 0.3636 | 0.12 | 0.414 | 0.29 | 0.3151 | 0.19 | 0.4444 |

Table S4 Results of multiple regression analyses investigating the relationship between EEG asymmetry indices and eating questionnaire measures for the eyes open condition. Note that the p-value threshold after Bonferroni correction for 8 separate regression analyses is 0.0063. CC – cognitive control, DI – disinhibition.

|  | Frontal | | | | | | | | | | | | Parietal | | | |
| --- | --- | --- | --- | --- | --- | --- | --- | --- | --- | --- | --- | --- | --- | --- | --- | --- |
|  | Alpha F3/F4 (n=99) | | Low alpha F3/F4 (n=102) | | Alpha F5/F6 (n=96) | | Low alpha F5/F6 (n=95) | | Alpha F7/8 (n=97) | | Low alpha F7/F8  (n=97) | | Alpha (n=83) | | Low alpha (n=84) | |
|  | Beta | p-value | Beta | p-value | Beta | p-value | Beta | p-value | Beta | p-value | Beta | p-value | Beta | p-value | Beta | p-value |
| CC | -0.10 | 1.0000 | -0.21 | 0.3043 | 0.45 | 0.0203 | 0.32 | 0.1470 | 0.43 | 0.0084 | 0.35 | 0.0525 | 0.17 | 0.1704 | 0.20 | 0.1460 |
| CC * gender | 0.14 | 0.6333 | 0.11 | 0.8431 | -0.36 | 0.0382 | -0.13 | 0.3080 | -0.32 | 0.0424 | -0.20 | 0.1733 | -0.29 | 0.1281 | -0.05 | 0.7840 |
| DI | 0.27 | 0.1287 | 0.21 | 0.1843 | 0.05 | 1.0000 | 0.00 | 1.0000 | -0.05 | 1.0000 | 0.04 | 0.6061 | 0.18 | 0.1372 | -0.04 | 0.9220 |
| DI * gender | -0.26 | 0.3269 | -0.16 | 0.3030 | 0.08 | 1.0000 | 0.10 | 0.5710 | 0.33 | 0.7059 | 0.20 | 0.4184 | -0.23 | 0.1292 | 0.05 | 0.9020 |
| Age | 0.02 | 1.0000 | 0.03 | 0.5811 | 0.00 | 1.0000 | 0.05 | 0.6190 | 0.12 | 0.2577 | 0.13 | 0.1889 | 0.19 | 0.0783 | 0.02 | 0.9410 |
| BMI | 0.19 | 0.0824 | 0.14 | 0.0774 | -0.15 | 0.1906 | -0.11 | 0.4480 | -0.05 | 0.7451 | -0.04 | 0.9804 | -0.10 | 0.9804 | -0.09 | 0.2860 |
| Gender | 0.37 | 0.0738 | 0.31 | 0.1793 | -0.16 | 0.2568 | -0.01 | 0.9800 | 0.39 | 0.1488 | 0.31 | 0.1938 | 0.17 | 0.2996 | 0.09 | 0.5620 |

Table S5 Results of multiple regression analyses investigating the relationship between EEG asymmetry indices and eating questionnaire measures for the eyes closed condition. Note that the p-value threshold after Bonferroni correction for 8 separate regression analyses is 0.0063. CC – cognitive control, DI – disinhibition.

|  | Frontal | | | | | | | | | | | | Parietal | | | |
| --- | --- | --- | --- | --- | --- | --- | --- | --- | --- | --- | --- | --- | --- | --- | --- | --- |
|  | Alpha F3/F4 (n=103) | | Low alpha F3/F4 (n=103) | | Alpha F5/F6 (n=100) | | Low alpha F5/F6 (n=100) | | Alpha F7/8 (n=93) | | Low alpha F7/F8  (n=94) | | Alpha (n=103) | | Low alpha (n=103) | |
|  | Beta | p-value | Beta | p-value | Beta | p-value | Beta | p-value | Beta | p-value | Beta | p-value | Beta | p-value | Beta | p-value |
| CC | -0.31 | 0.0836 | 0.08 | 0.5160 | 0.36 | 0.0501 | -0.01 | 0.9410 | 0.33 | 0.0854 | -0.03 | 0.9610 | 0.39 | 0.0354 | 0.33 | 0.1530 |
| CC * gender | 0.29 | 0.1121 | -0.13 | 0.4860 | -0.36 | 0.199 | 0.07 | 0.490 | -0.31 | 0.1310 | 0.10 | 0.9800 | -0.11 | 0.7059 | -0.21 | 0.4520 |
| DI | 0.04 | 0.5412 | 0.13 | 0.9610 | 0.07 | 0.5733 | 0.21 | 0.1620 | -0.02 | 1.0000 | 0.15 | 0.3510 | 0.01 | 1 | -0.06 | 1.0000 |
| DI * gender | 0.10 | 0.4138 | 0.00 | 0.980 | -0.17 | 0.2399 | -0.21 | 0.6380 | 0.10 | 0.5476 | -0.05 | 0.6670 | 0.09 | 0.9608 | -0.06 | 0.6670 |
| Age | -0.07 | 0.4013 | -0.07 | 0.7060 | 0.08 | 0.6667 | 0.02 | 0.8040 | 0.03 | 1.0000 | 0.15 | 0.5920 | -0.03 | 0.3511 | 0.03 | 0.8430 |
| BMI | 0.18 | 0.1135 | 0.16 | 0.1070 | -0.09 | 0.5942 | -0.12 | 0.2620 | -0.06 | 0.3004 | -0.03 | 0.8240 | -0.22 | 0.0402 | -0.11 | 0.2750 |
| Gender | 0.30 | 0.2711 | 0.30 | 1.0000 | -0.10 | 0.6863 | -0.13 | 1.0000 | 0.13 | 1.0000 | 0.10 | 1.0000 | 0.29 | 0.1279 | 0.11 | 0.6600 |

Table S6 Results of multiple regression analyses investigating the relationship between fMRI asymmetry indices (Sample 1) and eating behaviour. Note that the p-value threshold after Bonferroni correction for 6 separate regression analyses is 0.0083. The components have been ordered according to decreasing variance explained. CC – cognitive control; DI – disinhibition, RC – rotated component. Sample size n=106.

|  | RC1 | | RC2 | | RC3 | | RC5 | | RC4 | | RC6 | |
| --- | --- | --- | --- | --- | --- | --- | --- | --- | --- | --- | --- | --- |
|  | Beta | p-value | Beta | p-value | Beta | p-value | Beta | p-value | Beta | p-value | Beta | p-value |
| CC | -0.27 | 0.0821 | 0.25 | 0.8039 | 0.15 | 0.5942 | 0.09 | 1.0000 | 0.14 | 1.0000 | -0.15 | 0.5915 |
| CC * gender | 0.19 | 0.2468 | -0.14 | 0.3384 | -0.45 | 0.0162 | -0.24 | 0.6670 | -0.32 | 0.2010 | 0.32 | 0.1068 |
| DI | 0.11 | 1.0000 | -0.22 | 0.7451 | 0.11 | 0.2808 | -0.03 | 0.6380 | -0.12 | 0.5210 | 0.03 | 0.6863 |
| DI * gender | -0.08 | 1.0000 | 0.07 | 1.0000 | -0.19 | 0.1834 | 0.00 | 0.9800 | 0.18 | 0.7840 | -0.32 | 0.0574 |
| Age | -0.08 | 1.0000 | 0.06 | 0.6061 | -0.22 | 0.0271 | 0.06 | 0.3820 | -0.01 | 0.6380 | 0.04 | 0.5733 |
| BMI | -0.26 | 0.0487 | -0.18 | 0.0841 | 0.01 | 0.9216 | 0.00 | 1.0000 | 0.06 | 1.0000 | -0.04 | 0.8431 |
| Gender | 0.06 | 1.0000 | -0.34 | 0.0787 | 0.33 | 0.0857 | 0.08 | 0.7060 | 0.13 | 1.0000 | -0.29 | 0.3043 |

Table S7 Component loadings for each of the PCA’s rotated components (Sample 1) in the Three Factor Eating Questionnaire analysis. ROIs represent 12 regions of interest selected for the fMRI analyses. BA – Brodmann area, VTA – ventral tegmental area, NAcc – nucleus accumbens, ParacG – paracentral gyrus, PostcG – postcentral gyrus, ROI – region of interest, RC – rotated component.

| ROI | RC1 | RC2 | RC3 | RC5 | RC4 | RC6 |
| --- | --- | --- | --- | --- | --- | --- |
| BA44 | 0.32 | 0.41 | 0.15 | 0.46 | 0.29 | 0.29 |
| BA45 | -0.15 | 0.81 | 0.01 | 0.06 | 0.15 | 0.14 |
| BA6 | 0.26 | 0.75 | 0.00 | 0.03 | -0.21 | -0.10 |
| BA10 | 0.70 | 0.00 | 0.07 | -0.29 | 0.05 | 0.03 |
| BA9 | 0.81 | 0.20 | 0.02 | 0.08 | 0.19 | -0.02 |
| BA8 | 0.84 | -0.06 | 0.04 | 0.22 | -0.07 | -0.04 |
| BA46 | 0.73 | 0.04 | -0.33 | 0.03 | 0.05 | 0.09 |
| NAcc | 0.01 | 0.13 | 0.85 | -0.10 | 0.19 | 0.01 |
| VTA | 0.00 | 0.05 | -0.04 | 0.91 | 0.05 | -0.02 |
| BA7 | 0.10 | -0.05 | 0.06 | 0.10 | 0.87 | 0.06 |
| ParacG | 0.14 | 0.26 | -0.65 | -0.19 | 0.46 | -0.09 |
| PostcG | 0.02 | 0.04 | 0.03 | 0.01 | 0.04 | 0.98 |
| Cumulative variance  explained | 0.22 | 0.34 | 0.45 | 0.55 | 0.66 | 0.75 |

Table S8 Results of multiple regression analyses investigating the relationship between fMRI asymmetry indices (Sample 2) and eating behaviour. Note that the p-value threshold after Bonferroni correction for 6 separate regression analyses is 0.0083. The components have been ordered according to decreasing variance explained. CC – cognitive control, DI – disinhibition, RC – rotated component. Sample size n=86.

|  | RC1 | | RC6 | | RC2 | | RC3 | | RC4 | | RC5 | |
| --- | --- | --- | --- | --- | --- | --- | --- | --- | --- | --- | --- | --- |
|  | Beta | p-value | Beta | p-value | Beta | p-value | Beta | p-value | Beta | p-value | Beta | p-value |
| CC | 0.04 | 0.4732 | -0.07 | 0.5100 | -0.06 | 0.5217 | 0.02 | 0.8630 | 0.12 | 0.3530 | 0.07 | 0.5810 |
| CC * gender | -0.03 | 0.9804 | -0.12 | 0.4950 | 0.75 | 0.0319 | 0.01 | 1.0000 | -0.01 | 0.9410 | -0.02 | 1.0000 |
| DI | 0.22 | 0.0710 | 0.11 | 0.4520 | 0.08 | 0.4655 | -0.08 | 0.7450 | -0.05 | 0.7250 | 0.02 | 0.7450 |
| DI * gender | -0.11 | 0.7647 | 0.21 | 0.3060 | 0.48 | 0.1274 | 0.28 | 0.3640 | 0.26 | 0.2980 | 0.00 | 0.9410 |
| Age | 0.20 | 0.0805 | 0.12 | 0.4380 | 0.21 | 0.1300 | 0.03 | 0.4740 | 0.06 | 0.5940 | -0.09 | 0.6670 |
| BMI | 0.01 | 1.0000 | -0.01 | 1.0000 | -0.02 | 1.0000 | 0.02 | 0.8240 | -0.12 | 0.4700 | 0.04 | 0.7450 |
| Gender | 0.08 | 0.9020 | 0.07 | 0.8820 | 0.43 | 0.0776 | 0.35 | 0.2370 | 0.08 | 0.6190 | -0.19 | 0.3180 |

Table S9 Component loadings for each of the PCA’s rotated components (Sample 2) in the Three Factor Eating Questionnaire analysis. ROIs represent 12 regions of interest selected for the fMRI analyses. BA – Brodmann area, VTA – ventral tegmental area, NAcc – nucleus accumbens, ParacG – paracentral gyrus, PostcG – postcentral gyrus, ROI – region of interest, RC – rotated component.

| ROI | RC1 | RC2 | RC3 | RC5 | RC4 | RC6 |
| --- | --- | --- | --- | --- | --- | --- |
| BA44 | 0.71 | -0.13 | 0.05 | -0.06 | 0.22 | 0.16 |
| BA45 | 0.25 | 0.80 | -0.17 | -0.06 | 0.15 | -0.03 |
| BA6 | 0.18 | -0.04 | 0.17 | 0.82 | 0.21 | 0.17 |
| BA10 | -0.06 | 0.20 | 0.03 | 0.33 | 0.81 | -0.10 |
| BA9 | 0.70 | -0.03 | -0.14 | 0.11 | -0.27 | -0.37 |
| BA8 | 0.64 | 0.26 | 0.20 | 0.05 | 0.07 | 0.01 |
| BA46 | 0.31 | -0.27 | -0.09 | -0.35 | 0.65 | -0.04 |
| NAcc | -0.07 | 0.03 | -0.16 | 0.63 | -0.07 | -0.19 |
| VTA | 0.00 | 0.06 | -0.07 | -0.04 | -0.11 | 0.95 |
| BA7 | 0.05 | -0.14 | 0.86 | -0.07 | 0.09 | -0.03 |
| ParacG | -0.20 | 0.64 | 0.27 | 0.07 | -0.11 | 0.12 |
| PostcG | 0.09 | 0.38 | 0.64 | 0.06 | -0.15 | -0.03 |
| Cumulative variance  explained | 0.14 | 0.26 | 0.37 | 0.48 | 0.59 | 0.70 |

Table S10 Results of multiple regression analyses investigating the relationship between fMRI asymmetry indices (Sample 3) and eating behaviour. Note that the p-value threshold after Bonferroni correction for 6 separate regression analyses is 0.0083. The components have been ordered according to decreasing variance explained. CC – cognitive control, DI – disinhibition, RC – rotated component. Sample size n=140.

|  | RC1 | | RC6 | | RC2 | | RC3 | | RC4 | | RC5 | |
| --- | --- | --- | --- | --- | --- | --- | --- | --- | --- | --- | --- | --- |
|  | Beta | p-value | Beta | p-value | Beta | p-value | Beta | p-value | Beta | p-value | Beta | p-value |
| CC | 0.04 | 0.5476 | 0.05 | 0.4510 | -0.02 | 1.0000 | -0.01 | 1.0000 | 0.01 | 0.8040 | -0.01 | 0.7451 |
| CC * gender | -0.04 | 1.0000 | -0.01 | 1.0000 | 0.35 | 0.0510 | 0.17 | 0.2330 | 0.16 | 0.1650 | 0.11 | 0.5412 |
| DI | -0.01 | 0.7843 | 0.10 | 0.6330 | -0.01 | 0.8430 | -0.07 | 1.0000 | 0.08 | 0.3830 | 0.00 | 1.0000 |
| DI * gender | 0.00 | 1.0000 | -0.13 | 0.6860 | -0.26 | 0.1280 | 0.00 | 1.0000 | -0.20 | 0.9020 | -0.16 | 0.7255 |
| Age | -0.05 | 0.8627 | 0.06 | 0.5640 | -0.03 | 1.0000 | 0.05 | 1.0000 | -0.01 | 0.8040 | -0.04 | 0.7843 |
| BMI | 0.14 | 0.0716 | 0.12 | 0.3960 | 0.08 | 0.9610 | 0.02 | 1.0000 | -0.03 | 1.0000 | -0.15 | 0.0487 |
| Gender | 0.07 | 0.8431 | -0.08 | 0.3710 | -0.12 | 0.5410 | -0.11 | 0.4300 | -0.12 | 0.8040 | 0.26 | 0.4904 |

Table S11 Component loadings for each of the PCA’s rotated components (Sample 3) in the TFEQ analysis. ROIs represent 12 regions of interest selected for the fMRI analyses. BA – Brodmann area, VTA – ventral tegmental area, NAcc – nucleus accumbens, ParacG – paracentral gyrus, PostcG – postcentral gyrus, ROI – region of interest, RC – rotated component.

| ROI | RC1 | RC2 | RC3 | RC5 | RC4 | RC6 |
| --- | --- | --- | --- | --- | --- | --- |
| BA44 | 0.08 | 0.98 | 0.11 | 0.1 | 0.01 | 0.00 |
| BA45 | 0.97 | 0.09 | 0.12 | 0.01 | -0.06 | 0.08 |
| BA6 | 0.37 | -0.04 | 0.72 | 0.05 | -0.17 | 0.00 |
| BA10 | 0.03 | 0.22 | 0.59 | 0.32 | 0.13 | -0.01 |
| BA9 | 0.05 | 0.03 | 0.27 | 0.76 | 0.11 | -0.07 |
| BA8 | -0.03 | 0.14 | -0.05 | 0.84 | -0.02 | 0.03 |
| BA46 | 0.01 | 0.11 | 0.86 | -0.01 | 0.03 | 0.02 |
| NAcc | -0.11 | 0.04 | 0.03 | 0.12 | 0.88 | 0.08 |
| VTA | 0.16 | -0.05 | -0.04 | -0.14 | 0.17 | 0.84 |
| BA7 | -0.06 | 0.08 | 0.11 | 0.31 | -0.44 | 0.56 |
| ParacG | -0.97 | -0.09 | -0.13 | -0.01 | 0.05 | -0.07 |
| PostcG | -0.09 | -0.98 | -0.11 | -0.11 | -0.01 | 0.01 |
| Cumulative variance  explained | 0.17 | 0.34 | 0.49 | 0.62 | 0.71 | 0.79 |

# Supplementary Figures

Fig. S1 Correlation matrix of behavioural measures – Sample 1. * - p<0.05; ** - p<0.005; *** - p<0.0005


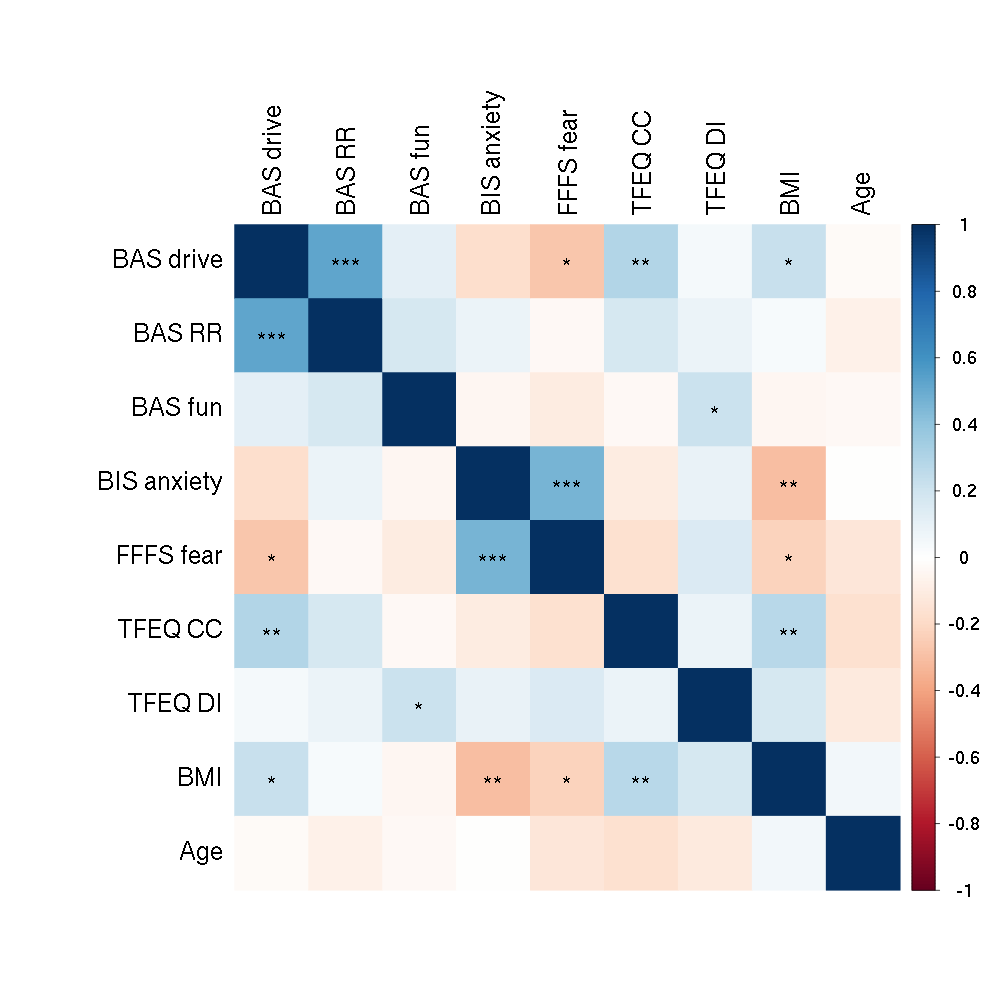


Fig. S2 Correlation matrix of behavioural measures – Sample 2. * - p<0.05; ** - p<0.005; *** - p<0.0005


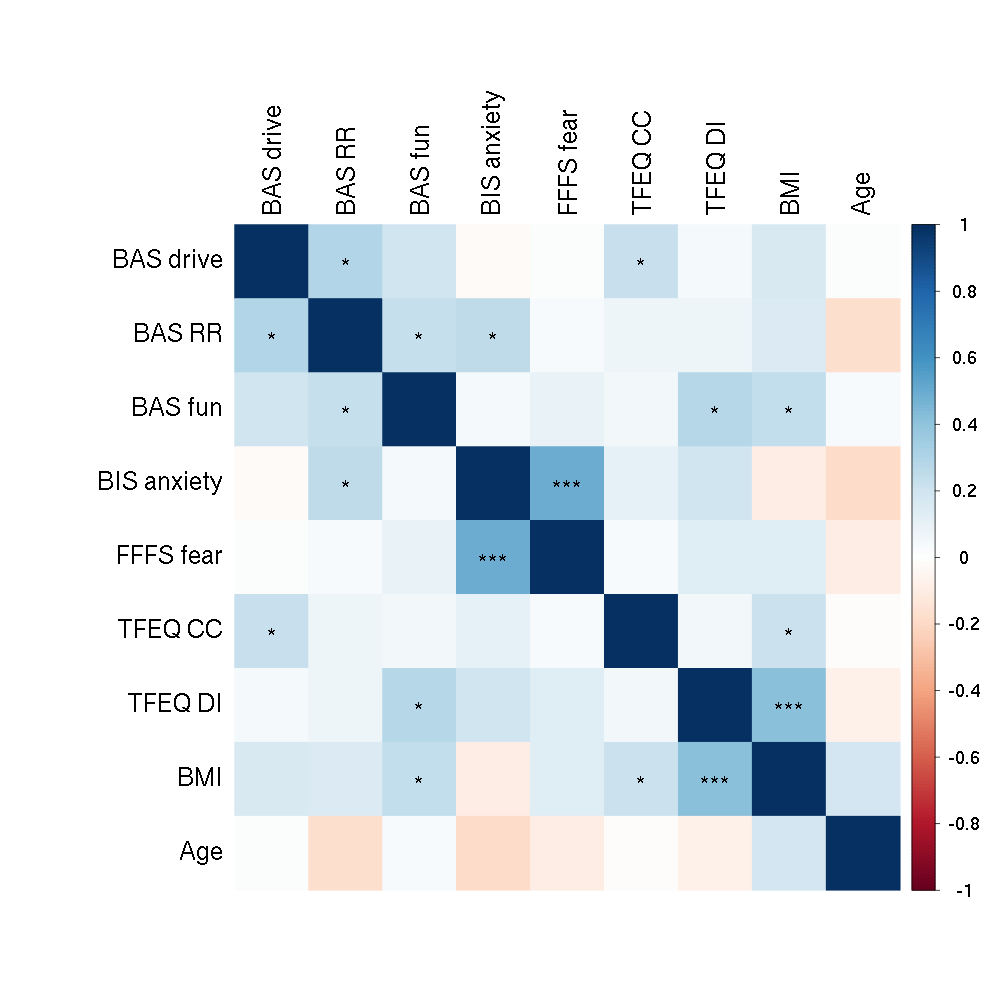


Fig. S3 Correlation matrix of behavioural measures – Sample 3. * - p<0.05; ** - p<0.005; *** - p<0.0005


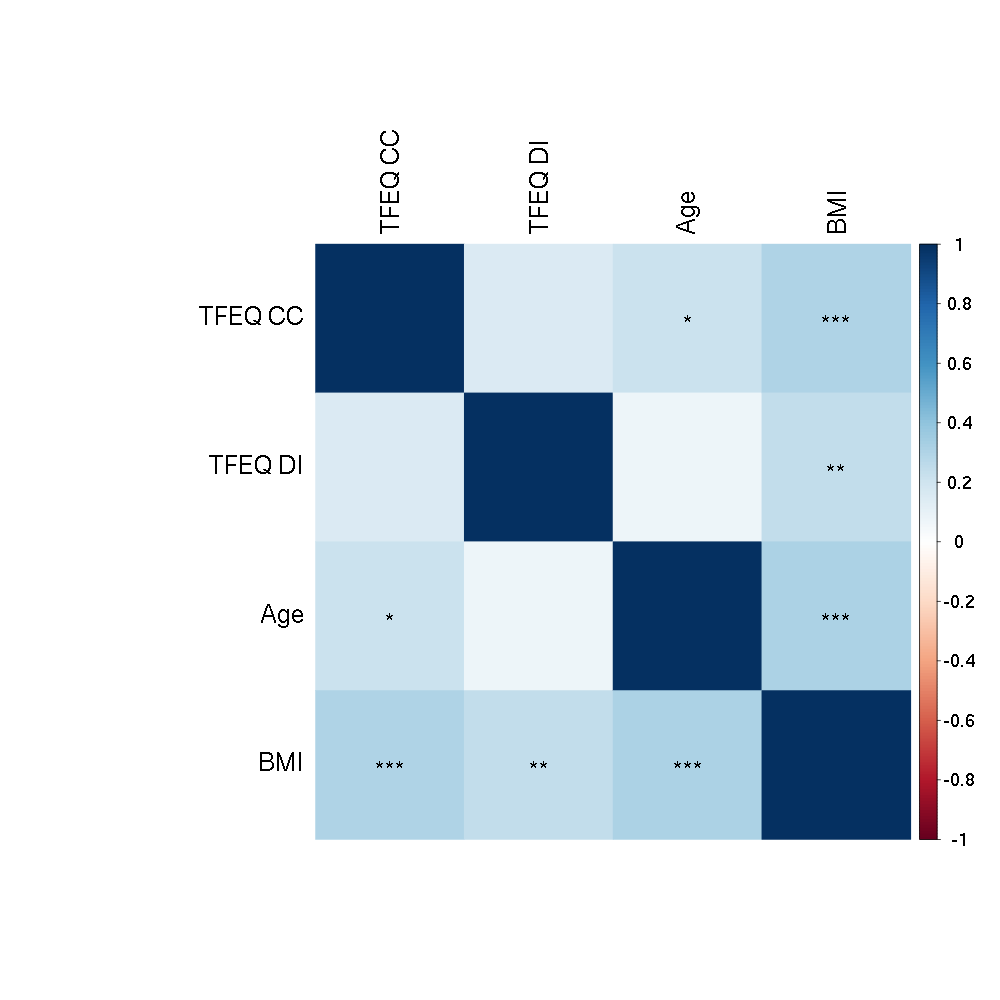

Supplement: Supplementary file 1 — Appendix S1: Supporting information [file HBM-41-1136-s001.docx]
